# Modulation of Water Vapor Sorption by Pore Engineering in Isostructural Square Lattice Topology Coordination Networks

**DOI:** 10.1021/acsami.4c06412

**Published:** 2024-06-20

**Authors:** Xia Li, Andrey A. Bezrukov, Wells Graham, Debobroto Sensharma, Xiang-Jing Kong, Timo Thonhauser, Michael J. Zaworotko

**Affiliations:** † Department of Chemical Science, Bernal Institute, University of Limerick, Limerick V94 T9PX, Republic of Ireland; ‡ Department of Physics and Center for Functional Materials, Wake Forest University, Winston–Salem, North Carolina 27109, United States

**Keywords:** water sorbents, hydrolytically stable, metal−organic materials, crystal engineering, pore chemistry

## Abstract

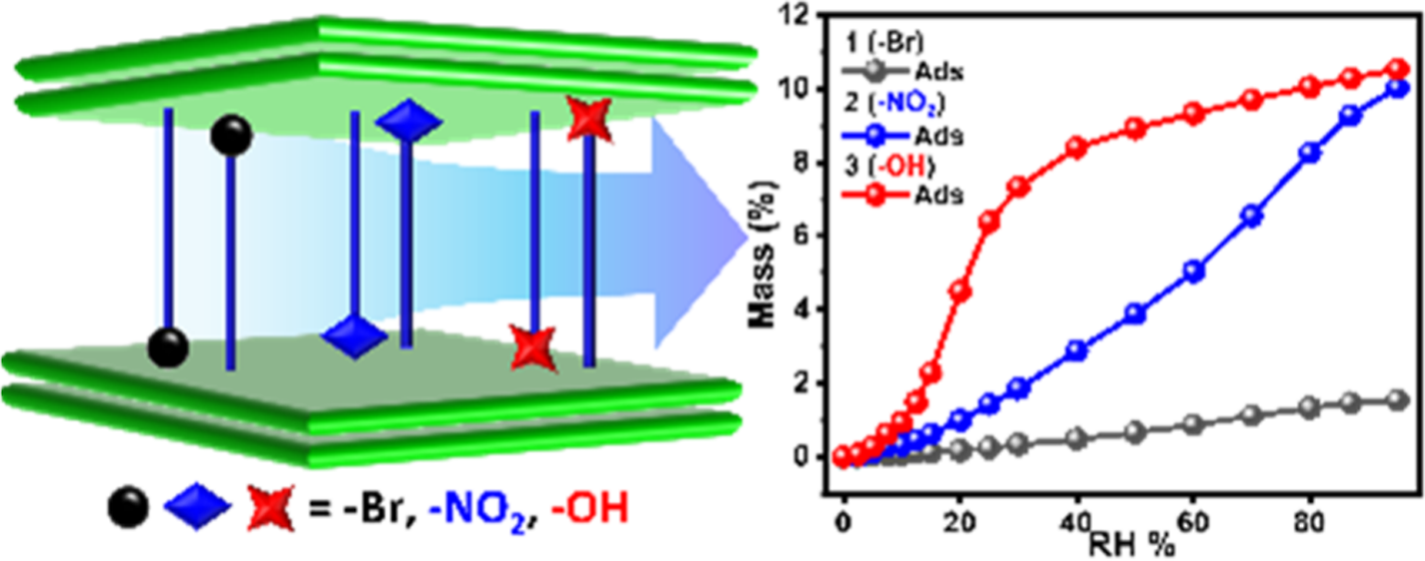

We report a crystal-engineering study conducted upon a platform of three mixed-linker square lattice (**sql**) coordination networks of general formula [Zn(Ria)(bphy)] [bphy = 1,2-bis(pyridin-4-yl)hydrazine, H_2_Ria = 5-position-substituted isophthalic acid, and R = –Br, –NO_2_, and –OH; compounds **1**–**3**]. Analysis of single-crystal X-ray diffraction data of **1**–**2** and the simulated crystal structure of **3** revealed that **1**–**3** are isomorphous and sustained by bilayers of **sql** networks linked by hydrogen bonds. Although similar pore shapes and sizes exist in **1**–**3**, distinct isotherm shapes (linear and S shape) and uptakes (2.4, 11.6, and 13.3 wt %, respectively) were observed. Ab initio calculations indicated that the distinct water sorption properties can be attributed to the R groups, which offer a range of hydrophilicity. Calculations indicated that the significantly lower experimental uptake in compound **1** can be attributed to a constricted channel. The calculated water-binding sites provide insights into how adsorbed water molecules bond to the pore walls, with the strongest interactions, water–hydroxyl hydrogen bonding, observed for **3**. Overall, this study reveals how pore engineering can result in large variations in water sorption properties in an isomorphous family of rigid porous coordination networks.

## Introduction

Porous crystalline metal–organic materials (MOMs),^1,2^ which are also known as metal–organic frameworks (MOFs)^3,4^ and porous coordination polymers (PCPs),^5,6^ are a class of materials that exhibit modular compositions, thereby enabling pore engineering (tunable pore shape, size, and chemistry) through crystal-engineering approaches.^1,7^ The inherent modularity of most MOMs^1,8^ enables pore engineering by placing chemical entities on the building blocks (nodes and/or linkers) of networks. In principle, the pore chemistry of MOMs^9^ can be engineered by precise control over the position and spatial arrangement of the chemical functionalities to target specific applications. Indeed, pore engineering of MOMs has been explored for utility in gas separation/storage,^10,11^ water sorption,^12,13^ catalysis,^14,15^ ion/electron transport,^16^ and energy-transfer^17^ applications.

Despite these promising properties of MOMs, the inherent hydrolytic instability of many MOMs, including several benchmark materials, means that they are unstable in aqueous media, limiting their practical utility,^18−20^ especially when it comes to water-related applications.^21^ Indeed, the deliberate design and synthesis of hydrolytically stable MOMs still poses a significant design challenge. To date, whereas >118,000 MOM structures have been deposited in the Cambridge Structural Database (CSD),^22^ relatively few examples are reported as water sorbents.^21,23^ Furthermore, among these known water sorbents, reports of systematic pore engineering to adjust water vapor sorption performance remain rare. Approaches to pore engineering in the context of water sorption can be classified into two approaches: ligand functionalization; node modification. For ligand functionalization, groups with variable hydrophobicity can be attached to the linkers, as exemplified by **CAU-10**;^24−26^**UiO-66**;^27^**MIL-101**;^12,28^**MIL-53**;^29^**MOF-303**;^30^**MOF-77**;^31^**Zn(NDI)-X**, X = H, NHEt, and Set;^32^ and **ZnF(TZ)**.^33^ For node modification, metal substitution [**M**_**2**_**Cl**_**2**_**(BTDD)**, M = Mn, Co, and Ni]^34^ or attachment of functional groups to metal clusters (**MOF-808**;^35^**BUT-46**^36^) can modify the pore chemistry and in turn water sorption properties. In these studies, the hydrophobic or hydrophilic groups that decorate pore walls were found to adjust water vapor sorption parameters such as water uptake and the isotherm profile.

**Figure 1:**
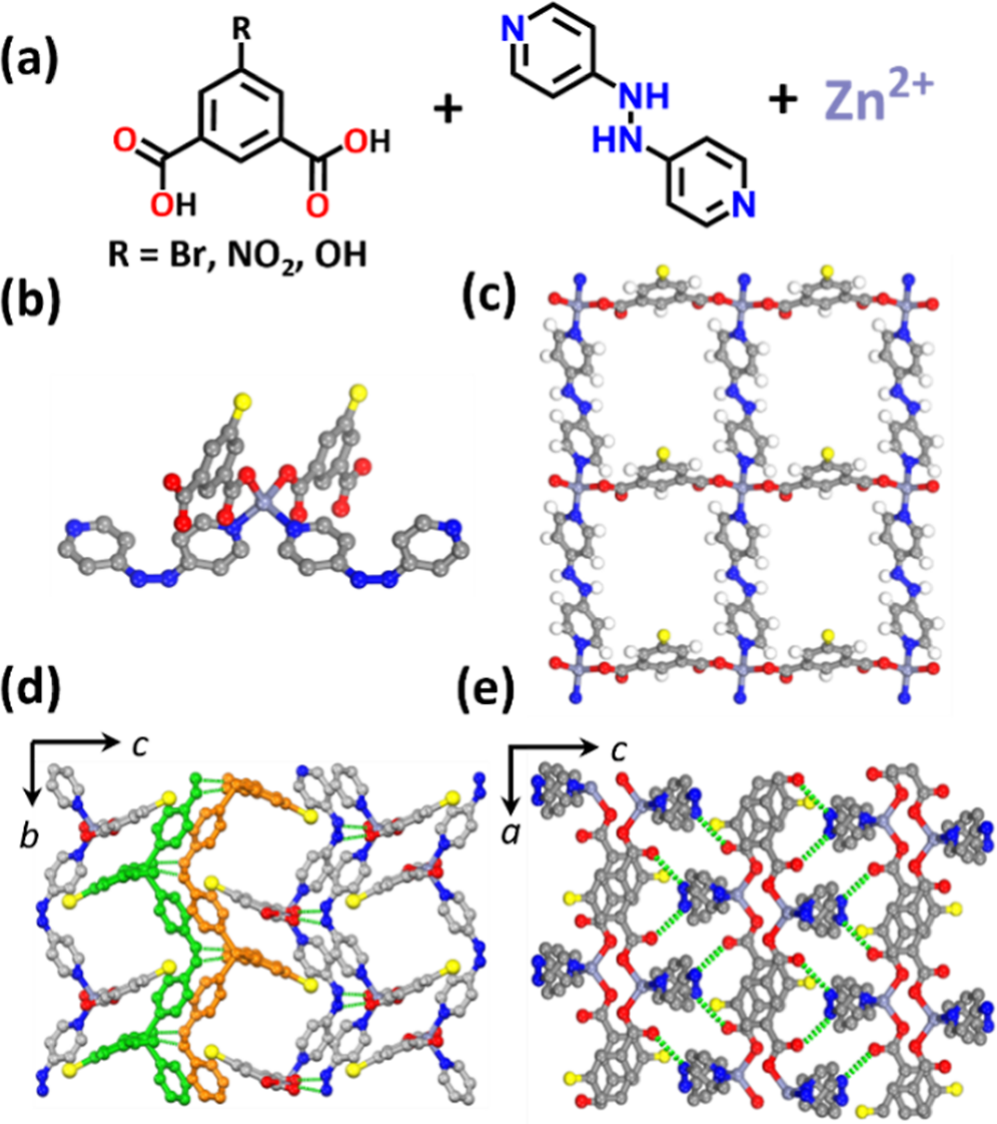
(a) Structures of ligands H_2_Ria R = –Br, –NO_2_, –OH, and bphy and the Zn^2+^ cation. (b) Coordination environment of the Zn^2+^ cation. (c) Simplified **sql** four lattices of [Zn(Ria)(bphy)] (R = –Br, –NO_2_, and –OH). (d) Interdigitated **sql** network with double layers (each layer was marked in green and orange) which is connected by interlayer hydrogen bonds (green dashed line) and 1D channels lies along the *a*-axis and (e) view along the *b*-axis (hydrogen atoms have been omitted for clarity). Color codes: N, blue; Zn, purple; H, white; C, gray; Br, yellow; and O, red.

Square lattice (**sql**) networks are the most commonly reported 2D coordination networks^37^ thanks in part to their amenability to crystal engineering through modularity of the metal node, linker ligands, and, if appropriate, anions.^38^ Even greater diversity can be offered by mixed-linker or “rectangular” **sql** networks,^39^ as two linkers in the structure can be varied. Our recent analysis of crystal structures deposited in the TOPOS TTO∩CSD databases^22,40^ revealed >1300 mixed-linker **sql** network examples based on N-donor and dicarboxylate linker ligands.^39^ Despite this number of structures, studies reporting water vapor sorption in **sql** networks are, to our knowledge, limited to only five examples: **{[Cu(bpy)**_**2**_**(5-H**_**2**_**sip)**_**2**_**]·(H**_**2**_**O)**_**6**_**}**_***n***_,^41^**CID-5/CID-6**,^42^**MCID-1**,^43^**sql-(1,3-bib)(ndc)-Ni**,^44^ and **sql-(azpy)(pdia)-Ni**.^45^ Herein, we report a crystal-engineering strategy that afforded a platform of three isostructural mixed-linker **sql** networks with similar pore shapes and sizes but different pore chemistry. Pore chemistry was tuned by changing the substituent groups at the 5 position of the isophthalate linkers in [Zn(bia)(bphy)] (**1**), the previously reported network [Zn(nia)(bphy)]^46^ (**2**), and [Zn(hia)(bphy)] (**3**) [bia^2–^ = 5-bromoisophthalate, nia^2–^ = 5-nitroisophthalate, hia^2–^ = 5-hydroxy isophthalate, and bphy = 1,2-di(pyridin-4-yl)hydrazine], respectively (Figure 1a). This tuning of the pore chemistry of **1**–**3** resulted in dramatic changes in both water vapor sorption uptake and the sorption isotherm profiles.

## Experimental Section

### Materials and General Method

Commercially available reagents were used as received without further purification. The ligand azpy was prepared according to a reported method.^47^ Single-crystal reflection data were collected on a Bruker D8 QUEST diffractometer equipped with a Cu Kα microfocus source (λ = 1.5406 Å) and a Photon 100 detector. Powder X-ray diffraction (PXRD) patterns were recorded on a PANalytical X’Pert MPD Pro (Cu Kα, λ = 1.5418 Å) with a 1D X’Celerator strip detector. Thermogravimetric analyses (TGAs) were performed under N_2_ using a TA Instruments Q50 system. Samples were loaded into aluminum sample pans and heated at 10 K min^–1^ from room temperature to 500 °C. Low-pressure (0–1 bar) CO_2_ sorption isotherms were measured using a Micromeritics 3Flex instrument. Water vapor sorption measurements were conducted using a Surface Measurement System DVS Adventure instrument for isotherm determination and DVS Intrinsic for adsorption/desorption cycling.

#### Syntheses of [Zn(bia)(bphy)] (**1**)

A mixture of Zn(NO_3_)_2_·6H_2_O (0.05 mmol, 14.9 mg), H_2_bia (0.05 mmol, 12.3 mg), and (E)-1,2-di(pyridin-4-yl)diazene (azpy) (0.05 mmol, 9.2 mg) in 2 mL of *N*,*N*-dimethylformamide (DMF) and 2 drops of sodium hydroxide water solution (0.1 M) was added to a 10 mL glass vial and heated to 105 °C for 1 day. After the vial was cooled to room temperature, the mother liquid was decanted, and the crystals were rinsed three times with DMF (3 mL × 3) (yield ca. 75% based on Zn). *v*_max_ (cm^–1^) = 3058, 1590, 1544, 1379, 1223, 1012, 833, 769, 714.

#### Single Crystals of [Zn(nia)(bphy)]^46^ (**2**)

The synthesis of **2** was similar to that of **1** except H_2_nia (0.05 mmol, 10.6 mg) was used instead of H_2_bia (yield ca. 79% based on Zn). *v*_max_ (cm^–1^) = 3521, 3237, 1668, 1613, 1342, 1200, 1021, 814, 723.

#### Microcrystalline Powder of [Zn(hia)(bphy)] (**3**)

The synthesis of **3** was similar to that of **1** except H_2_hia (0.05 mmol, 9.1 mg) was used instead of H_2_bia and bphy (0.05 mmol, 9.3 mg) was used instead of azpy (yield ca. 75% based on Zn). *v*_max_ (cm^–1^) = 3654, 3227, 1603, 1550, 1357, 1207, 1014, 740.

## Results and Discussion

### Synthesis and Structural Analysis

**Figure 2:**
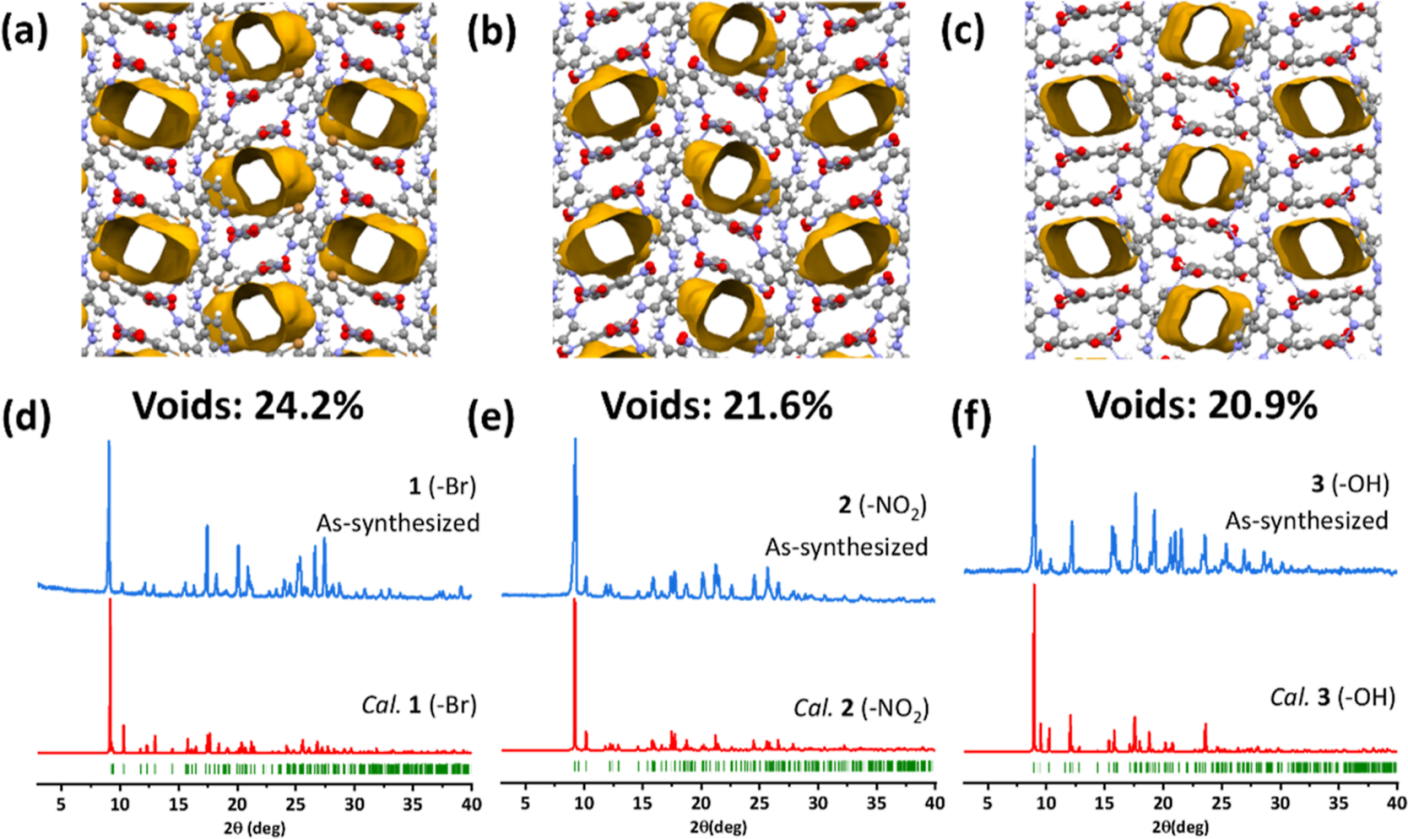
1D channels along the *a*-axis and void spaces of (a) **1**, (b) **2**, and (c) **3**. Comparison of experimental PXRD patterns of (d) **1**, (e) **2**, and (f) **3** and PXRD patterns calculated from the SCXRD-determined structures of **1**, **2**, and the simulated structure of **3**.

Single crystals of **1** and **2** were prepared by solvothermal reaction of precursor azpy [(*E*)-1,2-di(pyridin-4-yl)diazene] and H_2_Ria (5-R-isophthalic acid, R = bromo or nitro, respectively) in DMF and aqueous sodium hydroxide (0.1 M) at 105 °C. The reaction of azpy and H2Ria (R = hydroxyl) under the same conditions was unsuccessful for **3**, the PXRD pattern of the solid thereby obtained being different from those of **1** and **2** (Figure S1a). **3** was subsequently prepared as a microcrystalline powder by solvothermal reaction of bphy [1,2-di(pyridin-4-yl)hydrazine] and H_2_Ria (R = hydroxyl) under the same conditions. The PXRD pattern of **3** matched those of **1** and **2** (Figure S1b). Single-crystal X-ray diffraction (SCXRD) studies of **1** and **2** revealed **sql** topology and crystallization in monoclinic space group *P*2_1_/*n* (Figure 1 and Table S1). As previously reported, during the solvothermal synthesis of **1** and **2**, azpy was in situ reduced into bphy.^48^ Despite numerous attempts, single crystals of **3** could not be obtained. In order to determine the crystal structure of **3**, the –Br moiety of **1** was substituted to –OH in the crystal structure of **1**. The resulting structure was then optimized via ab initio relaxations to determine lattice parameters and atom positions. The PXRD pattern for the modeled crystal structure was then calculated and compared with experimental PXRD data. Good agreement between the calculated and experimental PXRD patterns was observed (Figures 2f and S2). The isomorphic nature of **1**–**3** was thereby confirmed (Tables S2 and S3).

The tetrahedral mononuclear molecular building blocks (MBBs)^49^ of **1**, **2**,^46^ and **3** are comprised of one Zn^2+^ cation coordinated to two N-donor atoms from different bphy ligands, two carboxylate O-donor atoms from different Ria^2–^ (R = bromo, nitro, and hydroxy) ligands (Figure 1b), and a general formula [Zn(Ria)(bphy)]. The torsion angles of the hydrazo moiety (C–N–N–C) in the bphy linkers of **1**–**3** were found to be 98.5(4)°, 95.9(3)°, and 103.6°, respectively (Table S4). **1**–**3** exhibit layered structures with **sql** topology; each rectangle is comprised of two parallel bphy ligands and two parallel isophthalate moieties that form the edges of a quadrilateral with Zn^2+^ cations at the vertices (Figure 1c). Similar lengths (vertical Zn–Zn distances) of the **sql** in **1** and **2** were observed along with the isophthalate-bridged edge [10.211(1) Å for **1** and 10.185(1) Å for **2**] and the bphy-bridged edge [11.228(1) Å for **1** and 11.104(1) Å for **2**, Table S4]. Interlayer hydrogen bonds were observed between hydrazo groups in bphy and uncoordinated O atoms of Ria^2–^ (R = bromo and nitro) ligands in neighboring **sql** layers [*d*_N...O_ = 2.832 (3), 2.861(4) Å; ∠_N–H...O_ = 154.0(2), 167.9(2)° for **1** and *d*_N...O_ = 2.864 (3), 2.857 (3) Å; ∠_N–H...O_ = 152.2(2), 171.2(2)° for **2**] which means that their structures can be described as bilayers of **sql** networks (Figure 1d,e and Table S4). Similar hydrogen bonds were observed in the simulated structure of compound **3**. These hydrogen-bonding interactions enable **sql** layers to form bilayers with functionalized groups oriented orthogonally in opposite directions. Adjacent bilayers were packed in an interdigitated manner (Figure 1d,e).

Because of the isomorphism of the three structures, their geometries are nearly identical and the pore sizes of their 1D channels are similar (5.2 × 6.2 Å for **1**, 5.2 × 6.1 Å for **2**, and 4.2 × 6.2 Å for **3**, Figure 2a–c). The substituent groups on the Ria linker did not significantly affect pore volumes, 24.2, 21.6, and 20.9% (probe size 1.2 Å, Figure 2a–c) for **1**–**3**, respectively. Furthermore, the experimental PXRD patterns of **1**–**3** match well with their calculated PXRD patterns from the SCXRD structures of **1**, **2**, and simulated structure of **3** (Figure 2d–f). TGA revealed 8–15% weight losses for **1**–**3** upon initial heating with no further weight losses until around 370 °C (Figure S3). Fourier transform infrared studies of **1**–**3** indicated the presence of guest water molecules in **2** and **3** with O–H water stretching peaks at 3521 and 3654 cm^–1^, respectively (Figure S4). Variable-temperature PXRD experiments were conducted on as-synthesized samples of **1**–**3** under a N_2_ atmosphere. During temperature ramping from 25 to 400 °C, PXRD data indicated retention of crystallinity (Figures S5–S7).

### Gas Sorption Studies

**Figure 3:**
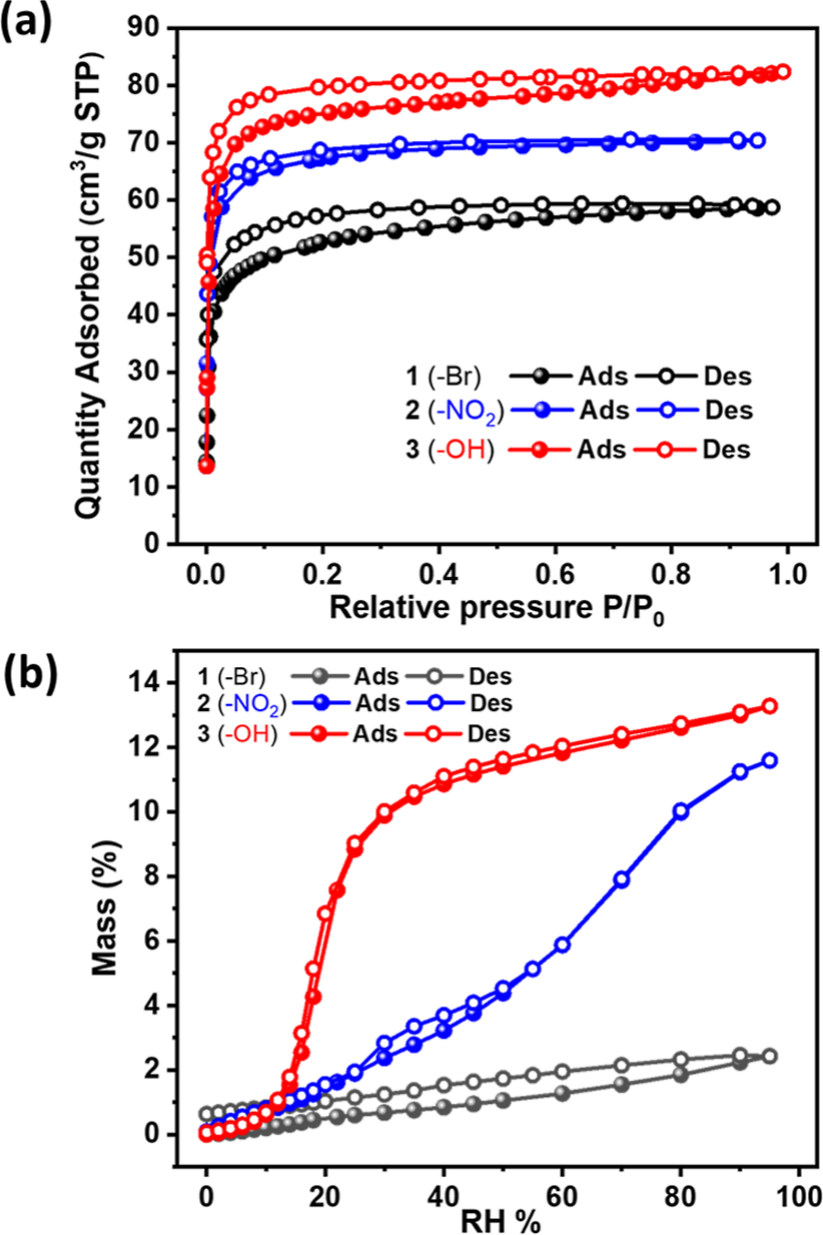
(a) CO_2_ sorption isotherms of **1**–**3** measured at 195 K. (b) Dynamic water vapor sorption isotherms of **1**–**3** at 298 K from 0 to 95% RH.

The as-synthesized samples were activated prior to gas sorption studies by exchanging with dichloromethane (DCM) daily for 3 days and heating under a dynamic vacuum at 60 °C for 12 h. The permanent porosity of **1**, **2**, and **3** was characterized by CO_2_ sorption isotherms collected at 195 K (Figure 3a). Type I isotherms and uptakes of 59, 70, and 82 cm^3^ g^–1^ at *P*/*P*_0_ = 0.95 for **1**, **2**, and **3** were observed, respectively. No significant hysteresis was observed during the desorption processes for **1**, **2**, and **3**. The type I isotherm shape observed for **1–3** is characteristic of rigid microporous materials and the order of CO_2_ uptakes correlated with the pore volume ranking. N_2_ sorption experiments at 77 K conducted on **1**–**3** did not show a significant uptake (Figure S8).

### Water Vapor Sorption Studies

Water vapor isotherms were measured from 0 to 95% RH at 298 K (Figure 3b). The diverse pore chemistries of **1**–**3**, with substituted Ria groups offering different degrees of hydrophobicity, enabled significant variation in their water sorption properties. **1** exhibited negligible water vapor uptake over the entire relative pressure range with 2.4 wt % at 95% RH, indicating strong hydrophobicity originating from the bromo group in **1**. Conversely, **2** exhibited a nearly linear water vapor isotherm with a maximum uptake of 11.6 wt %, which we attribute to the weaker hydrophobicity of the nitro group vs the bromo group. **3** exhibited a stepped water vapor sorption isotherm with little water uptake below RH = 10–20%, followed by a steep increase until maximum uptake reached 13.3 wt %. Two consecutive cycles of water vapor sorption on **1**–**3** exhibited consistent water uptake and isotherm shape (Figure S9). Stepped isotherms render a sorbent of potential utility for atmospheric water-harvesting applications.^50^ Water contact angle measurements on **1**–**3** verified their variable hydrophobicity. 87.453, 64.001, and 24.659° contact angles were observed at 0 s for **1**–**3**, respectively, which changed to 87.453, 0, and 0° at 40 s, respectively. These results indicate hydrophobicity as follows: **1** > **2** > **3** (Figure S10). This order is consistent with the water vapor sorption measurements. The correlation between the pore chemistry in the host frameworks and water vapor sorption properties is intuitive. For example, as for **1** and **2**, water vapor uptake decreased and the inflection point shifted to higher RH for **CAU-10-OH** vs **CAU-10-NO**_**2**_.^25^ In addition, bromo-functionalized **NKMOF-8-Br** exhibited poor water vapor sorption like **NKMOF-8-Me**.^51^ A computational study^52^ predicted that reduced water uptake was reported for **UiO-66-Br** when compared to **UiO-66-OH**. These results are consistent with the trend observed in **1** and **3**.

**Figure 4:**
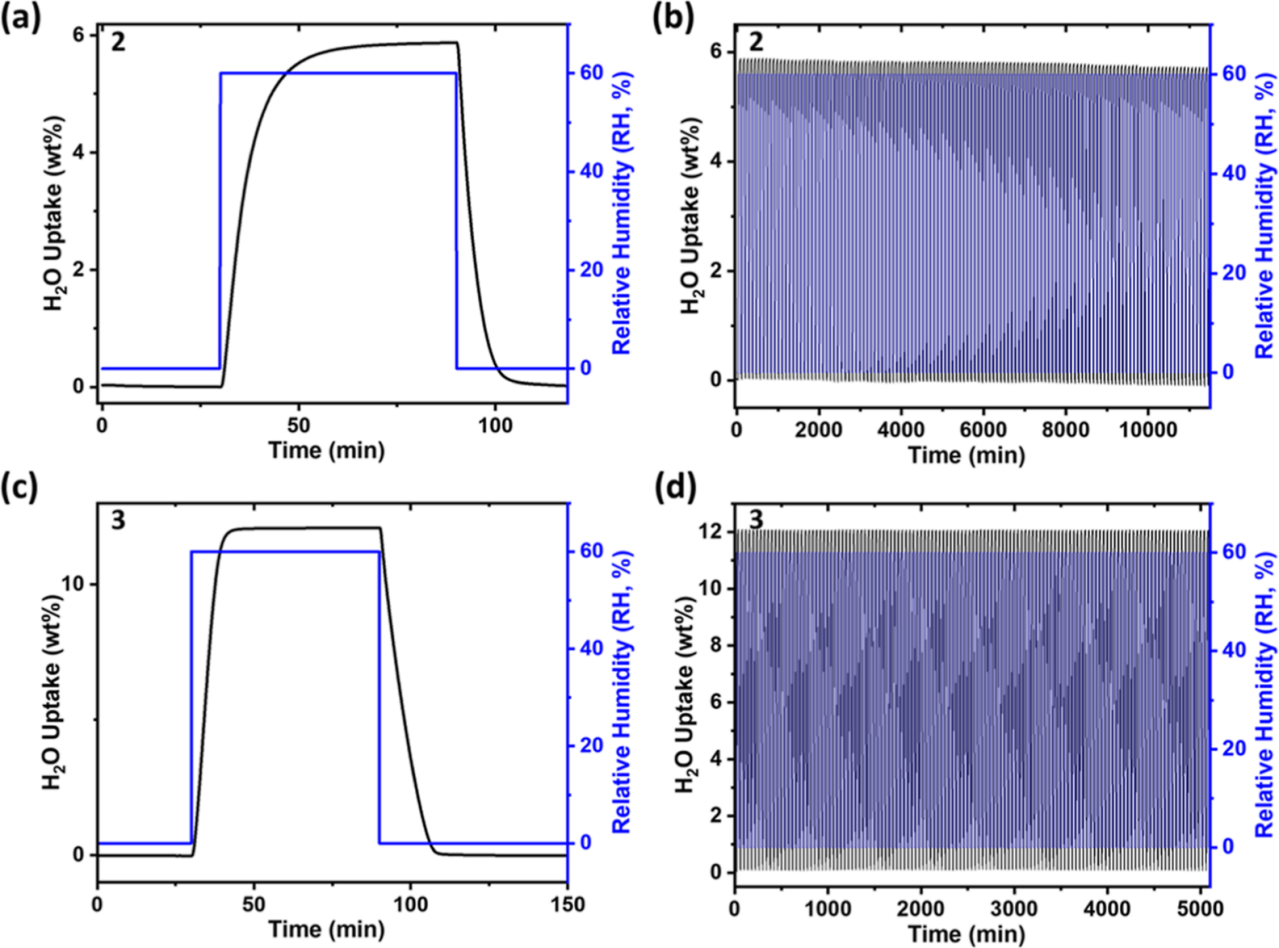
Dynamic adsorption–desorption water sorption of **2** (15.1 mg) after (a) 1 and (b) 128 cycles and **3** (13.6 mg) after (c) 1 and (d) 128 cycles from 0 to 60% RH at 298 K.

Unlike some MOMs which offer good performance with respect to gas sorption but suffer from structural collapse after exposure to water vapor, e.g., **MOF-5**,^18^**HKUST-1**,^20^ and **Co(bdp)**,^19^**1**–**3** were found to be kinetically stable in water. Experimental PXRD patterns of **1**–**3** revealed that activation (heating at 60 °C under vacuum), water exposure (soaking in water under ambient conditions for 24 h) and water adsorption–desorption cycles did not affect PXRD peak intensities or positions compared to their as-synthesized experimental PXRD patterns (Figures S11–S13). CO_2_ sorption at 195 K was conducted on samples of **1**–**3** after water sorption. No significant change of CO_2_ uptake or isotherm shape was observed (Figure S14). Water vapor sorption cycling experiments conducted on **2** and **3** also indicated good hydrolytic stability and recyclability. Indeed, **2** and **3** were subjected to 120 cycles without significant working capacity loss (Figures 4b,d and S15). Furthermore, temperature swing cycling experiments (adsorption at 198 K; desorption at 333 K) were conducted. Negligible decreases of uptakes during temperature swing cycling were observed, which revealed the good hydrolytic stability of **2** and **3** (Figure S16). The PXRD patterns of **2** and **3** after 128 cycles at 298 K indicated retention of crystallinity (Figure S17).

Sorption kinetics on **2** and **3** collected from 0 to 60% RH swing experiments conducted at 298 K are distinct, with the differences in kinetics reflecting the corresponding sorption isotherms. This is consistent with our recently reported isotherm-based kinetic model.^50^ Specifically, **3** exhibited an S-shaped isotherm and followed pseudo-“zero-order” (straight line) kinetics, while **2**, which has a linear isotherm, has a pseudo-“first-order” kinetic profile (Figure 4a,c). The isotherm-based kinetic model predicts faster adsorption for **3** than that for **2**, as was observed experimentally. Specifically, for comparable sample masses, **3** (adsorption = 12 min; desorption = 18 min) exhibited faster adsorption kinetics than **2** (adsorption = 32 min; desorption = 14 min) during the 0–60% RH swing process (Figure 4a,c). The kinetics data for **2** and **3** was fitted using our sorption isotherm-based kinetic model (Figures S18 and S19, see fitting details in Supporting Information). The physical interpretation of the model is limitation of sorption kinetics by water diffusion to the sorbent bed.^50^

### Structural Insights and Computational Studies

Understanding water sorption processes is valuable for gaining insights into water-binding sites. DFT calculations were performed to understand the mechanism of the water adsorption of **1**–**3**. The optimal binding locations within each structure are presented in Figures S20 and S22. The binding energies for water were calculated to be 41.4, 42.3, and 47.4 kJ mol^–1^ for **1**–**3**, respectively. In addition, diffusion barriers for a single adsorbate were calculated for each structure. The respective barriers for **1**, **2**, and **3** are 11.8, 10.2, and 8.8 kJ mol^–1^ (Figure S23). The order of the calculated binding energies and diffusion barriers agrees with the experimental isotherm order (Figure 3b).

**Figure 5:**
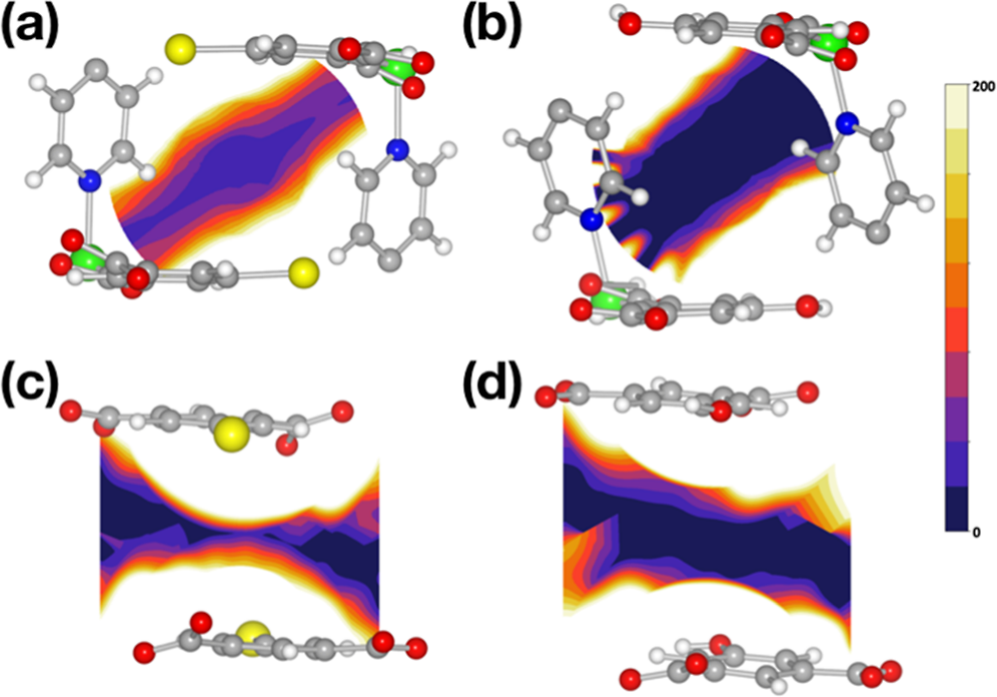
Potential energy landscape of water in **1** and **3**. (a) **1** pore axis view, (b) **3** pore axis view, (c) **1** cross pore view, and (d) **3** cross pore view. The planes shown are defined in Supporting Information Figure S24; the color bar is in kJ/mol. The possible diffusion channel for water in **1** is significantly narrower and more energetically unfavorable than that in **3**. See Figures S25 and S26 for more details. Color code: *N* = blue; Zn = green; H = white; C = gray; Br = yellow; and O = red.

In order to fully assess the hydrophobic effect of the various functional groups, we sampled two orthogonal planes between the different substituent groups; the planes are outlined and defined in Figure S24. The results for **3** and **1** are presented in Figure 5, and all other systems are in Figures S25 and S26. Figure 5 clearly shows the hydrophobic effect of the bromine moiety. The channel available for water to diffuse through is narrow and energetically unfavorable for **1**, whereas it is wide and energetically favorable in **3**. The bottleneck created by the bromine atoms, along with its hydrophobicity, effectively suppresses water diffusion, accounting for the negligible water uptake observed experimentally (Figure 3b).

## Conclusions

We report an isomorphic family of Zn-based **sql** networks with a similar pore shape and size but a different affinity to water vapor. The distinct water vapor sorption properties exhibited by **1**–**3** were influenced by substituents on the isophthalate linkers that provide various degrees of hydrophobicity. This platform of **sql** materials thereby allowed us to systematically and rationally fine-tune the water sorption properties. Specifically, three very different water sorption profiles were observed: hydrophobic, **1**; moderately hydrophilic, **2**; and pore filling via a stepped isotherm, **3**. Furthermore, these isotherms are entirely rational based upon the nature of the substituent in the Ria linkers. This work therefore provides a feasible design principle to obtain water sorbents with tunable water uptake and hydrophilicity, especially those with stepped isotherms that are desirable for water-harvesting applications. In short, pore engineering can be exploited to control water–pore vs water–water interactions. This work also demonstrates how even small changes in composition can profoundly impact water sorption properties in isomorphic sorbents.

## Supporting Information Available

Details of synthetic procedures, PXRD data, SCXRD data, TGAs, sorption isotherms, and simulation of structures (PDF)
X-ray data for [Zn(bia)(bphy)] (**1**) (CIF)

## Supplementary Material

am4c06412_si_001.pdf

am4c06412_si_002.cif
