# Supplementary material for: Modulation of Water Vapor Sorption by Pore Engineering in Isostructural Square Lattice Topology Coordination Networks
Source: ACS Appl Mater Interfaces. 2024 Jun 20;16(26):34402–8. doi: 10.1021/acsami.4c06412 (PMC11232023; doi:10.1021/acsami.4c06412)
Supplement: Supplementary file 1 — am4c06412_si_001.pdf [file am4c06412_si_001.pdf]

Supporting Information

Modulation of Water Vapor Sorption by Pore Engineering in Isostructural sql Topology Coordination Networks

Xia Li,† Andrey A. Bezrukov,† Wells Graham,‡ Debobroto Sensharma,† Xiang-Jing Kong,† Timo Thonhauser,‡ Michael J. Zaworotko†*

†Department of Chemical Science, Bernal Institute, University of Limerick, Limerick, V94 T9PX, Republic of Ireland
‡Department of Physics and Center for Functional Materials, Wake Forest University, Winston-Salem, North Carolina 27109, United States

* Email: michael.zaworotko@ul.ie

Contents

1. Materials and Synthesis S2
2. Single-crystal X-ray diffraction measurements. S2
3. Thermogravimetric analysis (TGA) S3
4. IR spectra Fourier Transform Infrared (FTIR) Spectroscopy S3
5. Powder X-ray diffraction measurements S3
6. Variable Temperature Powder X-ray Diffraction (VT-PXRD) S3
7. Gas sorption measurements S4
8. Dynamic vapor sorption (DVS) experiments S4
9. Computational simulation S5
10. Water contact angle S6
11. Supporting Figures and Tables S7
12. Reference S37

1. Materials and Synthesis

All chemicals 5-bromoisophthalic acid (H_2bia), 5-nitroisophthalic acid (H_2nia), 5-hydroxyisophthalic acid (H_2hia), and $Zn(NO_3)_2·6H_2O$ were obtained commercially and used as received without further purification. Synthesis ligand (*E*)-1,2-di(pyridin-4-yl)diazene was accomplished by applying previously reported procedures.[1]

Single crystal of [Zn(bia)(bphy)] (**1**):
Synthesis: A mixture of $Zn(NO_3)_2·6H_2O$ (0.05 mmol, 14.9 mg), H_2bia (0.05 mmol, 12.3 mg), and (*E*)-1,2-di(pyridin-4-yl)diazene (azpy) (0.05 mmol, 9.2 mg) in 2 mL of *N, N*-dimethylformamide (DMF) and 2 drops of sodium hydroxide water solution (0.1 M) was added to a 10mL glass vial and heated to 105 °C for 1 day. After the vial was cooled to room temperature, the mother liquid was decanted, and the crystals were rinsed three times with DMF (3 mL × 3, yield, ~ 75% based on Zn). ν_{max} (cm^{-1}) = 3058, 1590, 1544, 1379, 1223, 1012, 833, 769, 714.

Single crystal of [Zn(nia)(bphy)][2] (**2**):
Synthesis: The synthesis of **2** was similar to that of **1** except H_2nia (0.05 mmol, 10.6 mg) was used instead of H_2bia (yield, ~ 79% based on Zn). ν_{max} (cm^{-1}) = 3521, 3237, 1668, 1613, 1342, 1200, 1021, 814, 723.

Microcrystalline powder of [Zn(hia)(bphy)] (**3**):
Synthesis: The synthesis of **3** was similar to that of **1** except H_2hia (0.05 mmol, 9.1 mg) was used instead of H_2bia and bphy (0.05 mmol, 9.3 mg) were used instead of azpy (yield, ~ 75% based on Zn). ν_{max} (cm^{-1}) = 3654, 3227, 1603, 1550, 1357, 1207, 1014, 740.

2. Single-crystal X-ray diffraction measurements.

Single-crystal reflection data were collected on a Bruker Quest diffractometer equipped with a CMOS detector and IµS microfocus X-ray source (Cu K_α, λ = 1.54178 Å; Mo K_α, λ = 0.71073 Å). Indexing was performed using APEX3[3] (Difference Vectors method). Absorption correction was performed by a multi-scan method implemented in SADABS.[4] Space group was determined using XPREP implemented in APEX3.[3] Structural solution and refinement against F^2 were carried out using the SHELXL non-linear least squares implemented in Olex2 v1.2.10.[5,6] All non-hydrogen framework atoms were refined with anisotropic parameters, while H atoms were placed in calculated positions and refined using a riding model. Some of the disordered atoms have been refined isotropically. All the crystals were measured under liquid N_2 flow at a temperature of 100K to avoid the phase transformation caused by guest molecules escaping in the air. Crystallographic data and structural refinement information are listed in Tables S1. The structure of phases [Zn(bia)(bphy)] (**1**) was solved and refined

in the $P2_1/n$ space group. Crystallographic data for the structures reported in this paper have been deposited with the Cambridge Crystallographic Data Centre as supplementary publication No. CCDC 2244649.

3. Thermogravimetric analysis (TGA)

Thermogravimetric analyses (TGA) were performed under N_2 using a TA Instruments Q50 system. Samples were loaded into aluminium sample pans and heated at 10 K min^{-1} from room temperature to 500 °C.

4. IR spectra Fourier Transform Infrared (FTIR) Spectroscopy

Spectra were obtained by using a FTIR spectrometer (Agilent technologies, Cary 630) in the range of wavelength 4000-650 cm^{-1}.

5. Powder X-ray diffraction measurements

Powder X-ray diffraction patterns were recorded on a PANalytical X'Pert MPD Pro (Cu Ka, λ = 1.5418 Å) with a 1D X'Celerator strip detector. Experiments were conducted in continuous scanning mode with the goniometer in the theta-theta orientation. Incident beam optics included the Fixed Divergences slit with anti-scatter slit PreFIX module, with a 1/8°divergence slit and a 1/4° anti-scatter slit, as well as a 10 mm fixed incident beam mask and a Soller slit (0.04 rad). Divergent beam optics included a P7.5 anti-scatter slit, a Soller slit (0.04 rad), and a Ni β filter. The data were collected in the range of 2θ = 3 – 40°. Raw data were then evaluated using the X'Pert HighScore Plus™ software V 4.1 (PANalytical, The Netherlands).

6. Variable Temperature Powder X-ray Diffraction (VT-PXRD)

Diffractograms at different temperatures were recorded using a PANalytical X'Pert Pro-MPD diffractometer equipped with a PIXcel3D detector operating in scanning line detector mode with an active length of 4 utilizing 255 channels. Anton Paar TTK 450 stage coupled with the Anton Paar TCU 110 Temperature Control Unit was used to record the variable temperature diffractograms. The diffractometer is outfitted with an Empyrean Cu LFF (long fine focus) HR (9430 033 7300x) tube operated at 40 kV and 40 mA and CuKα radiation (λ_α = 1.54056 Å) was used for diffraction experiments. Continuous scanning mode with the goniometer in the theta-theta orientation was used to collect the data. Incident beam optics included the Fixed Divergences slit, with a 1/4° divergence slit and a Soller slit (0.04 rad). Divergent beam optics included a P7.5 anti-scatter slit, a Soller slit (0.04 rad), and a Ni-β filter. In a typical experiment, 20 mg

of sample was ground into a fine powder and loaded on a zero-background sample holder made for Anton Paar TTK 450 chamber. The data were collected from 4 - 40° (2θ) with a step size of 0.0167113° and a scan time of 50 seconds per step. Crude data were analyzed using the X'Pert HighScore Plus™ software V 4.1 (PANalytical, The Netherlands). The sample was heated up to 523 K.

7. Gas sorption measurements

CO_2 and N_2 sorption isotherms were measured using Micromeritics 3Flex instrument. Dichloromethane (DCM) exchanged [Zn(bia)(bphy)] (**1**), [Zn(nia)(bphy)] (**2**), [Zn(hia)(bphy)] (**3**) were degassed under high vacuum at 60 °C for 10 h on Micromeritics Smart VacPrep instrument. The activated sample (ca. 100 mg) was transferred to 3Flex and evacuated at room temperature for 10 hours before the measurements.

8. Dynamic vapor sorption (DVS) experiments

Water vapor sorption isotherms were measured on ca. 10 mg sample using a Surface Measurement Systems Adventure Dynamic Vapor Sorption (DVS) system which gravimetrically measures the uptake and loss of vapor using air as a carrier gas. Pure water was used as the adsorbate for these measurements and temperature was maintained at 298 K by enclosing the system in a temperature-controlled incubator. The mass of the sample was determined by comparison to an empty reference pan and recorded by a high-resolution microbalance with a precision of 0.01 µg. Sorption isotherms were measured from 0 to 95% RH stepwise with a convergence equilibrium criterion dm/dt = 0.01 %/min. The minimum and maximum equilibration times for each step were 10 and 360 min, respectively. The samples were exchanged with DCM and heated to 100 °C for 2h in situ before water sorption.

Water vapor sorption kinetics was measured using a Surface Measurement Systems Intrinsic Dynamic Vapor Sorption (DVS) system which gravimetrically measures the uptake and loss of vapor using air as a carrier gas. Pure water was used as the adsorbate for these measurements and temperature was maintained at 298 K. The mass of the sample was determined by comparison to an empty reference pan and recorded by a high-resolution microbalance with a precision of 0.1 µg. Adsorption at 60 % RH and desorption at 0 % RH was performed for specified time and number of cycles.

Experimental sorption kinetics was modelled using isotherm-based kinetics model recently published by us.[7] Adsorption and desorption kinetics was modelled using Eqn. 1. RH_{bed} was determined from adsorption branch of the isotherm at the corresponding uptake. Three parameters were fitted: isotherm scaling factor, k and t0, where t0 was

fitted in 0-3 minutes range.

$$\frac{dw}{dt} = k \cdot (RH_{flow} - RH_{bed}) \quad \textbf{S1}$$

where w is uptake (wt.%), k is sorption coefficient, RH_{flow} is relative humidity in the flow and RH_{bed} is relative humidity in the sample bed.

9. Computational simulation

Ab initio simulations were performed at the density functional theory (DFT) level using the Vienna Ab Initio Simulation Package (VASP)[8,9]. In order to capture the van der Waals interactions between the host material and guest molecules, we utilized the vdW-DF[10–13] functional. To find the optimized structures, we allowed the unit cell volume along with all atomic positions to relax freely. The plane-wave energy cutoff was set to 600 eV with a SCF loop convergence of 0.01 meV, and only the gamma point was sampled in the Brillion zone. The calculations were considered fully converged once the magnitude of the forces on each atom were at or below 5 meV Å^{-1}. Binding energies were determined via the following equation:

$$\boldsymbol{E_{bind} = E_{MOF+water} - E_{MOF} - E_{water}} \quad \textbf{S2}$$

Induced charge densities were also calculated to determine the charge redistribution during the bond formation. This is calculated through similar means as binding energy except using charge density rather than total energy. Induced charge density plots allow for better understanding of the interactions between guest and host. Finally, a transition-state search algorithm (cNEB)[14] was used to determine the diffusion properties of water within the MOFs.

To further sample the chemical environment created by the substituent groups, two orthogonal planes between the groups were sampled using single-point SCF calculations. The planes are oriented down the pore axis and across the pore as seen in Figure S19. For each plane the water molecule was rotated around a chosen axis at varying distances in order to sweep out a sufficient portion of the planes. 144 and 185 calculations were carried out for plane 1 and 2, respectively within each system. Barycentric interpolation was then used to smoothen the contour lines.

To understand the dynamical properties of adsorption, we calculated the diffusion barrier of a single water molecule for each structure. The barriers of **1**, **2** and **3** are 11.8, 10.2 and 8.8 kJ mol^{-1}, respectively, as seen in Figure S23. The rate of diffusion can be related to the Arrhenius equation $A = Ce^{-\frac{E_i}{k_b T}}$, where A is the diffusion rate, C is a system dependent pre-factor, and E_i is the barrier energy. We can estimate the relative speeds of diffusion at T = 298K by taking ratios of diffusion rates for two systems. Approximating that the system-dependent pre-factors are equivalent, we find

$$\frac{A_3}{A_2} = e^{-\frac{(E_3 - E_2)}{k_b T}}, \quad \textbf{S3}$$

resulting in a ratio of 1.8 for **3** over **2**, in qualitative agreement with value of 2.7 observed for experimental kinetics at macro-scale (32 min adsorption time for **3** and 12 min adsorption time for **2**). When comparing **3** and **1** we see a ratio of 3.4, showing the significantly improved kinetics in **3**.

10. Water contact angle

Water contact angles were measured by using a Contact Angle Goniometer (L2004A1) produced by Ossila BV.

11. Supporting Figures and Tables

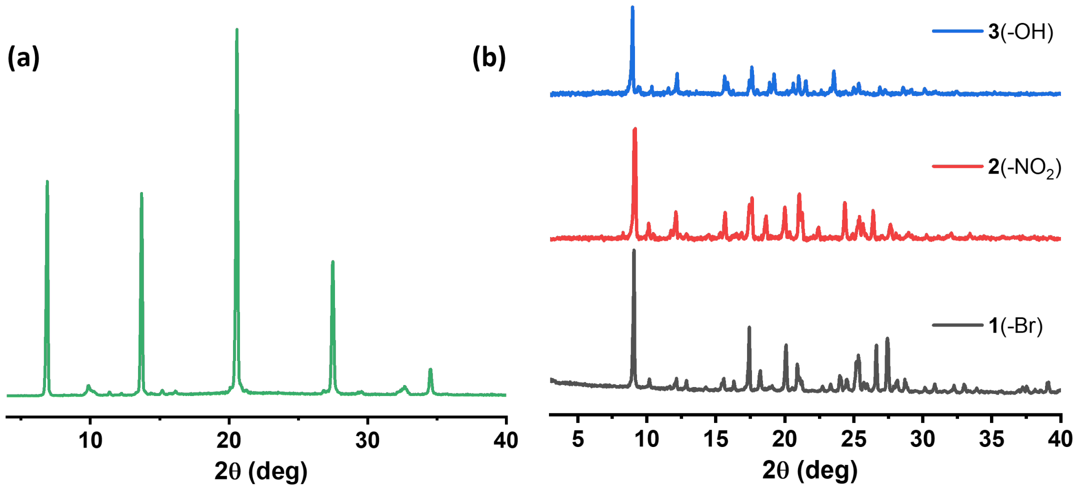

Figure S1. (a) PXRD patterns of the product that obtained under the condition of azpy ((*E*)-1,2-di(pyridin-4-yl)diazene) and H_2hia (5-hydroxy isophthalic acid) with *N, N*-dimethylformamide (DMF) and sodium hydroxide water solution (0.1 M) at 105 °C. (b) PXRD patterns of [Zn(bia)(bphy)] (**1**), [Zn(nia)(bphy)] (**2**), and [Zn(hia)(bphy)] (**3**).

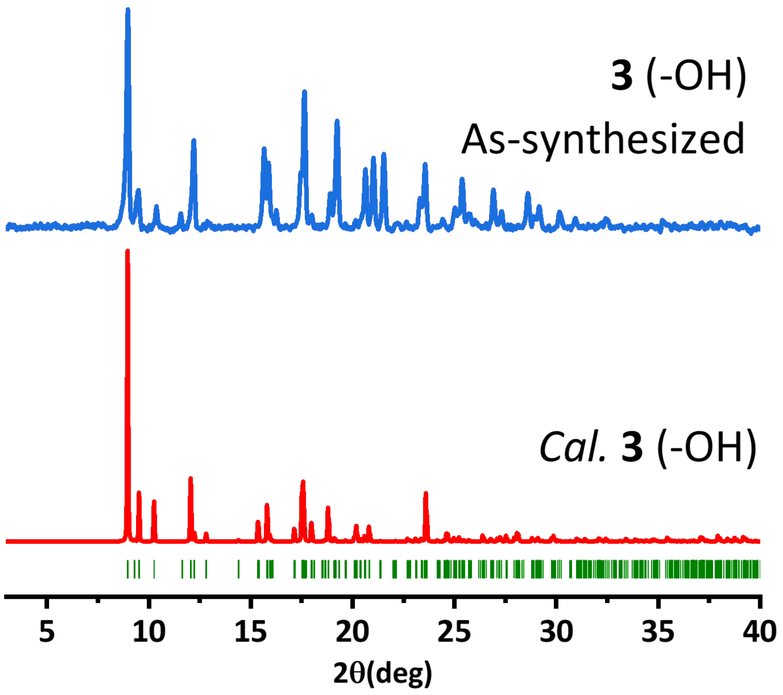

Figure S2. Experimental (blue), calculated PXRD patterns (red) and Bragg position (green) of **3** from its simulated structure.

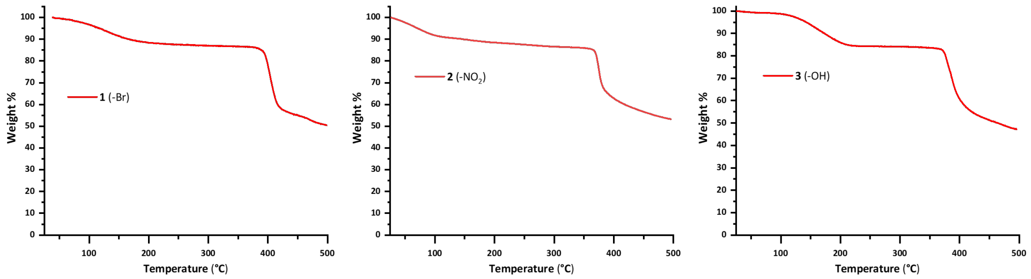

Figure S3. Thermogravimetric analysis of as-synthesized samples: [Zn(bia)(bphy)] (**1**), [Zn(nia)(bphy)] (**2**) and [Zn(hia)(bphy)] (**3**).

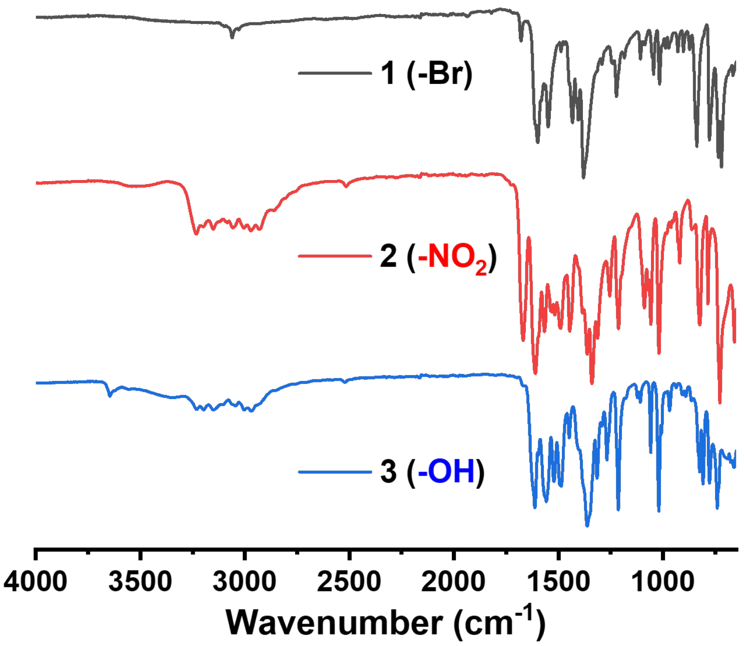

Figure S4. FT-IR spectra of as-synthesized samples of **1**, **2** and **3**.

$[Zn(bia)(bphy)]_n$ (**1**), IR: ν_{max} (cm^{-1}) = 3058, 1590, 1544, 1379, 1223, 1012, 833, 769, 714.

$[Zn(nia)(bphy)]_n$ (**2**), IR: ν_{max} (cm^{-1}) = 3521, 3237, 1668, 1613, 1342, 1200, 1021, 814, 723.

$[Zn(hia)(bphy)]_n$ (**3**), IR: ν_{max} (cm^{-1}) = 3654, 3227, 1603, 1550, 1357, 1207, 1014, 740.

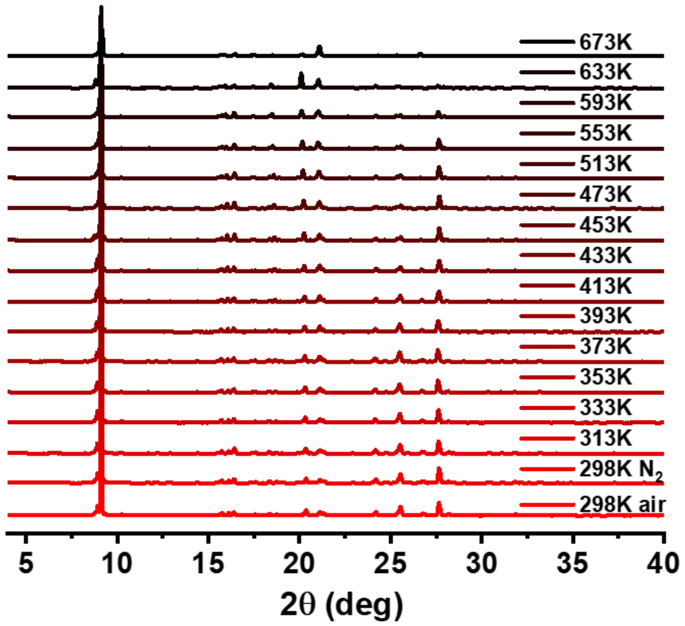

Figure S5. VT-PXRD patterns of [Zn(bia)(bphy)] (**1**) (conducted from an as-synthesized sample of **1** under N_2 flow).

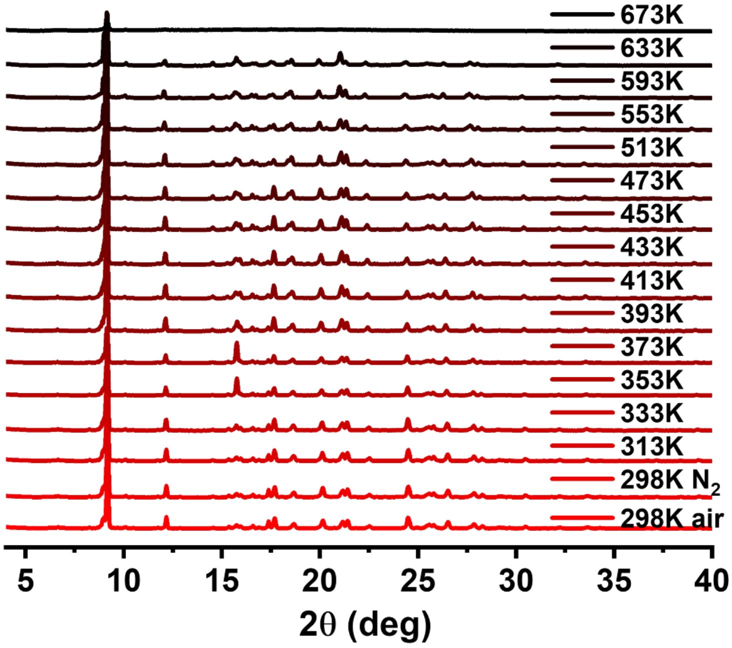

Figure S6. VT-PXRD patterns of [Zn(nia)(bphy)] (**2**) (conducted from an as-synthesized sample of **2** under N_2 flow).

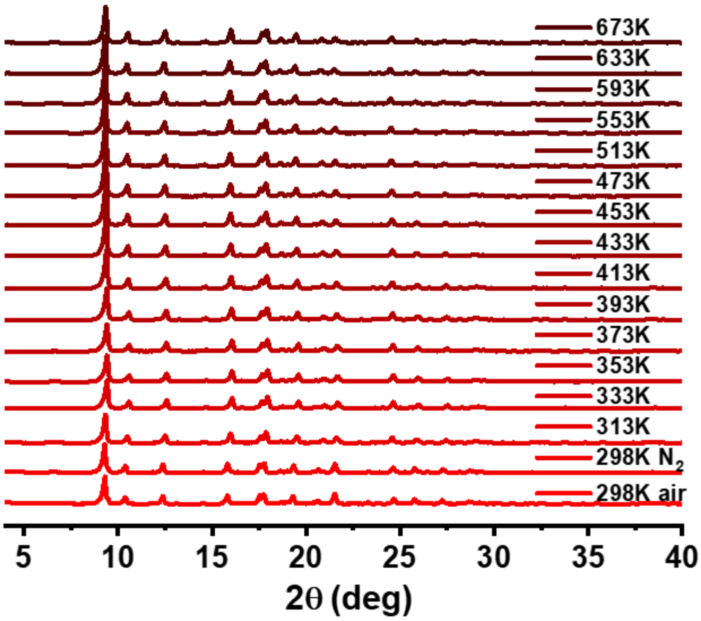

Figure S7. VT-PXRD patterns of [Zn(hia)(bphy)] (**3**) (conducted from an as-synthesized sample of **3** under N_2 flow).

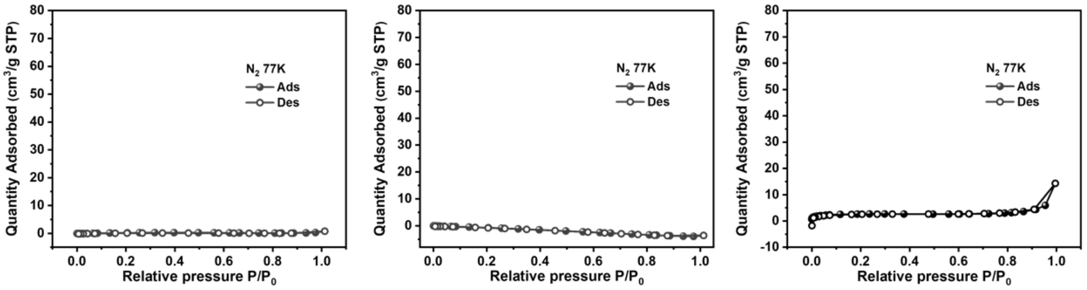

Figure S8. N_2 sorption isotherms of **1**, **2** and **3** measured at 77K.

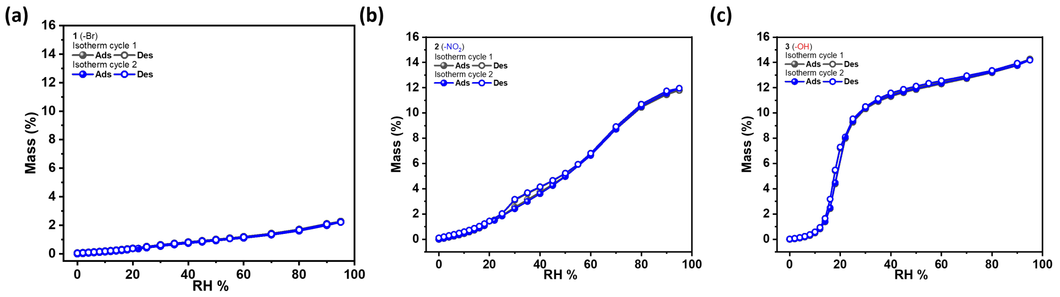

Figure S9. Two consecutive water vapor isotherms collected for (a) compound **1**, (b) compound **2** and (c) compound **3**.

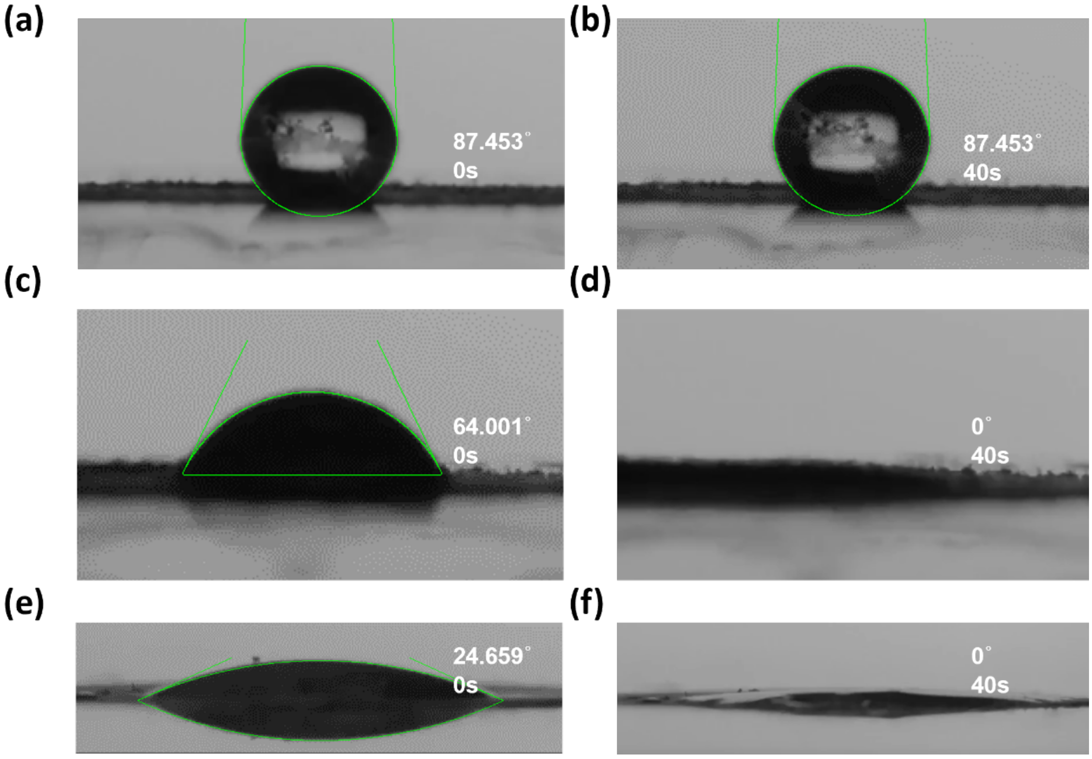

Figure S10. Water contact angle measured on compounds **1** at (a) 0s, (b) 40s; **2** at (c) 0s, (d) 40s; **3** at (e) 0s, (f) 40s.

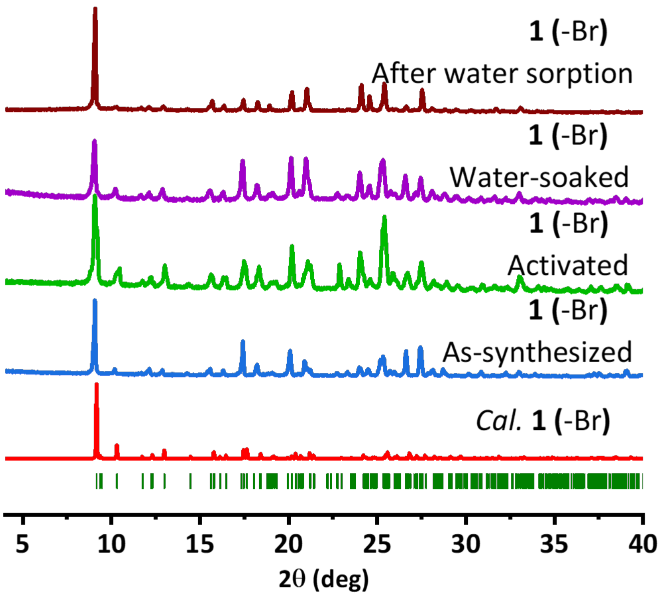

Figure S11. Calculated PXRD pattern of [Zn(bia)(bphy)] (**1**) from SCXRD data and experimental PXRD patterns of as-synthesized, activated, water-soaked and after water vapor sorption samples.

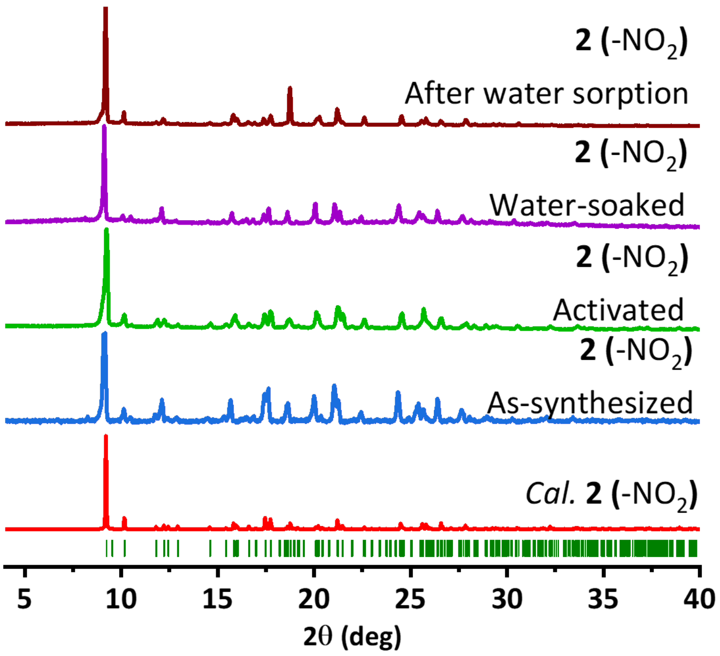

Figure S12. Calculated PXRD pattern of [Zn(nia)(bphy)] (**2**) from SCXRD data and experimental PXRD patterns of as-synthesized, activated, water-soaked and after water vapor sorption samples.

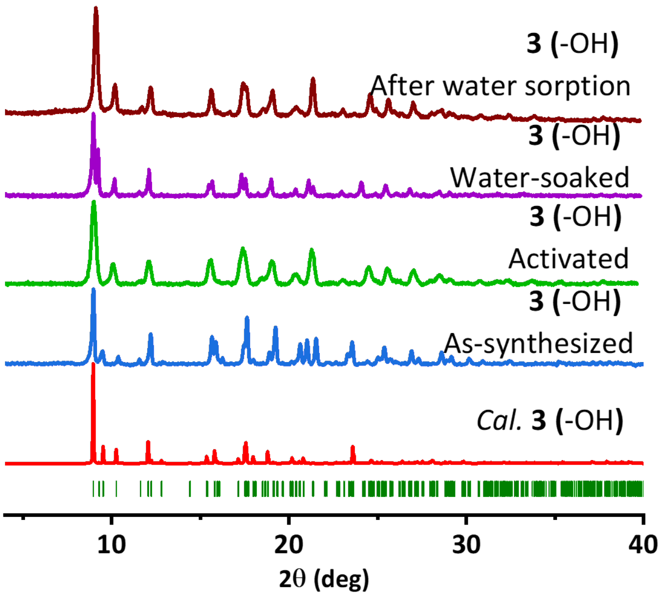

Figure S13. Calculated PXRD pattern of [Zn(hia)(bphy)] (**3**) from SCXRD data and experimental PXRD patterns of as-synthesized, activated, water-soaked and after water vapor sorption samples.

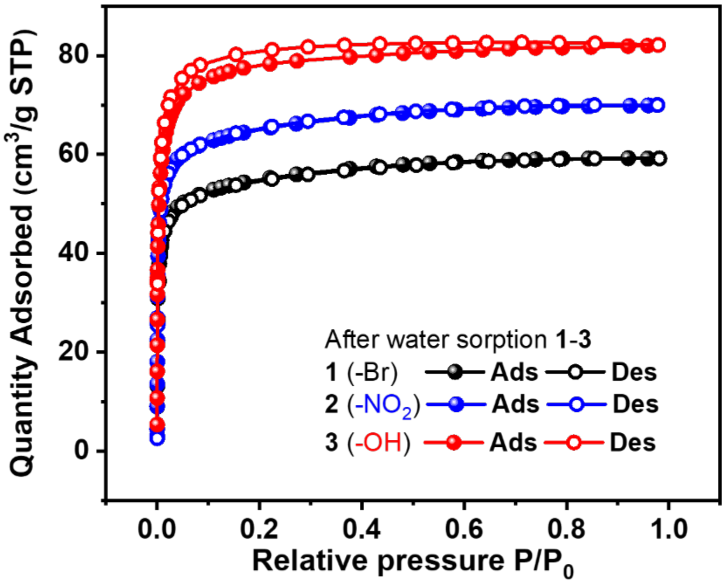

Figure S14. CO_2 sorption isotherms of **1**-**3** after water sorption at 195 K.

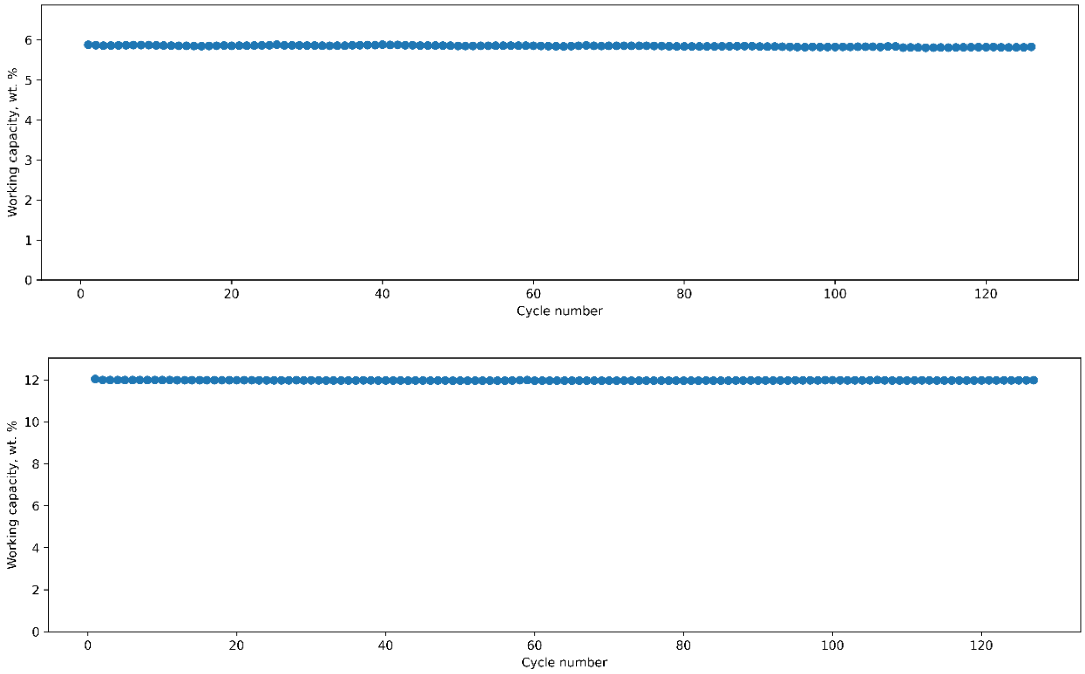

Figure S15. Working capacity as a function of cycle number for **2** (top) and **3** (bottom) in 0 – 60% RH cycling experiment at 298K.

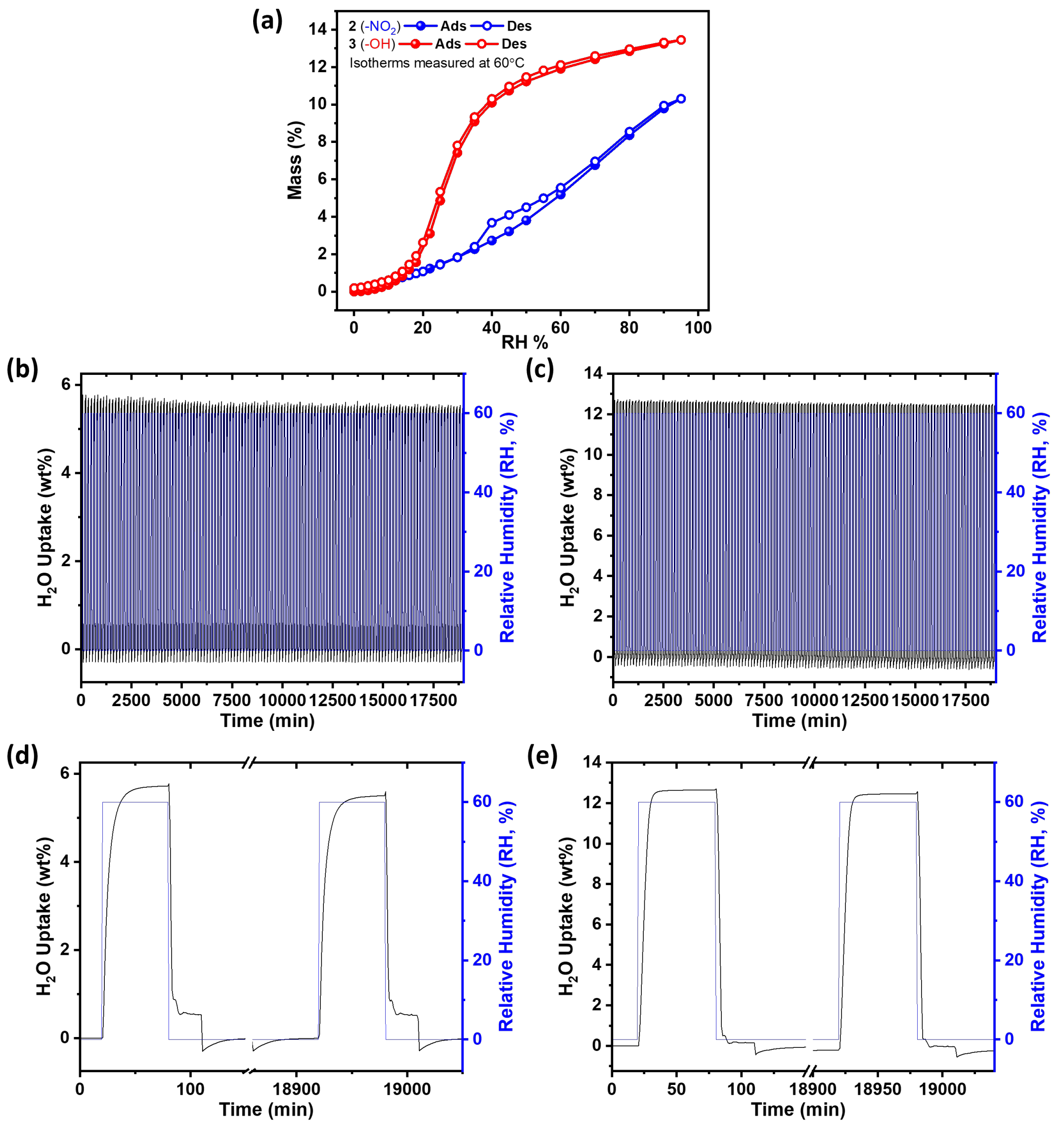

Figure S16. (a) Water vapor sorption isotherms of **2** and **3** at 333K; temperature swing cycling results, 298K for adsorption and 333K for desorption, of (b) **2** and (c) **3** from 0-60%RH for 128 cycles, respectively. The first and the last cycle of water vapor sorption for (d) **2** and (e) **3**, respectively.

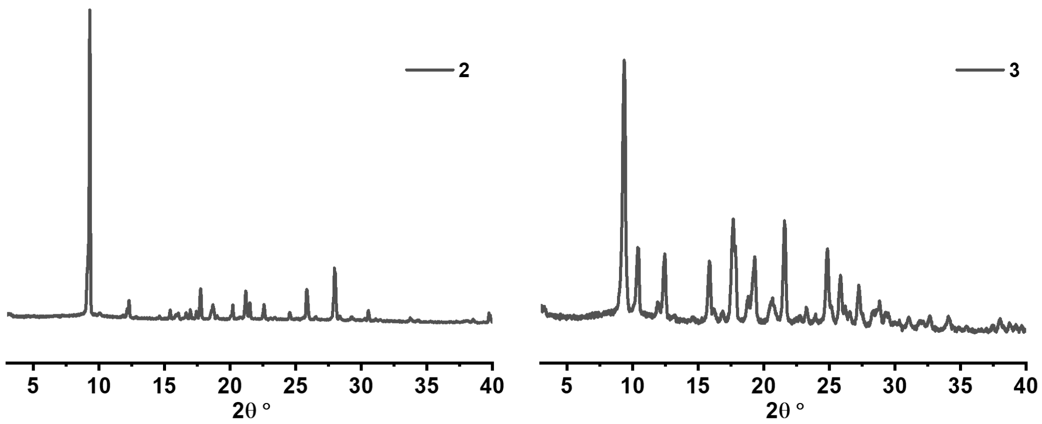

Figure S17. PXRD patterns of **2** and **3** after water cycling experiment at 298K.

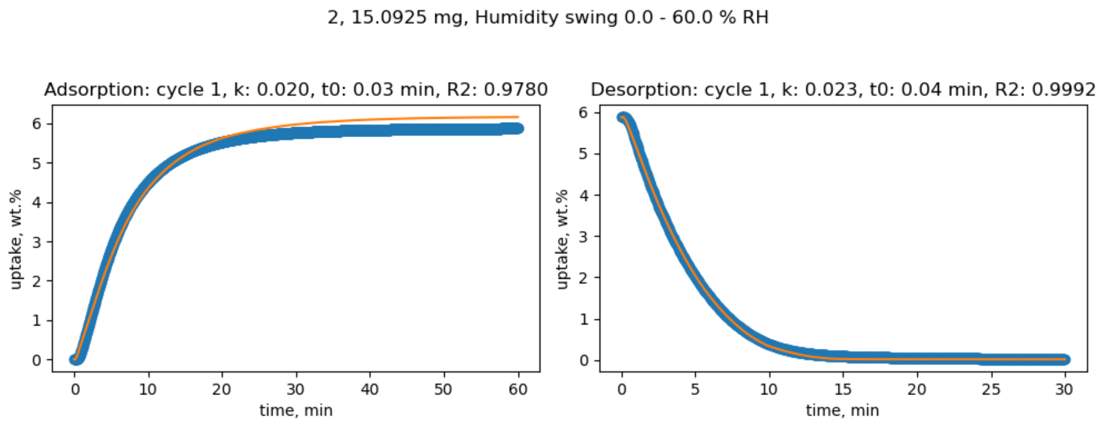

Figure S18. Fit of 0 – 60 %RH humidity swing kinetics data (blue) at 298 K on **2** (15.1 mg sample) using isotherm-based kinetics model (orange).

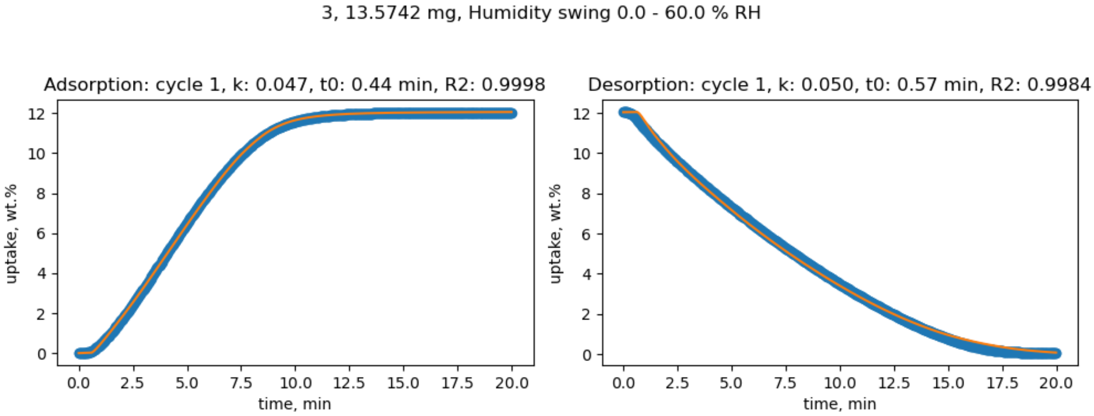

Figure S19. Fit of 0 – 60 %RH humidity swing kinetics data (blue) at 298 K on **3** (13.6 mg sample) using isotherm-based kinetics model (orange).

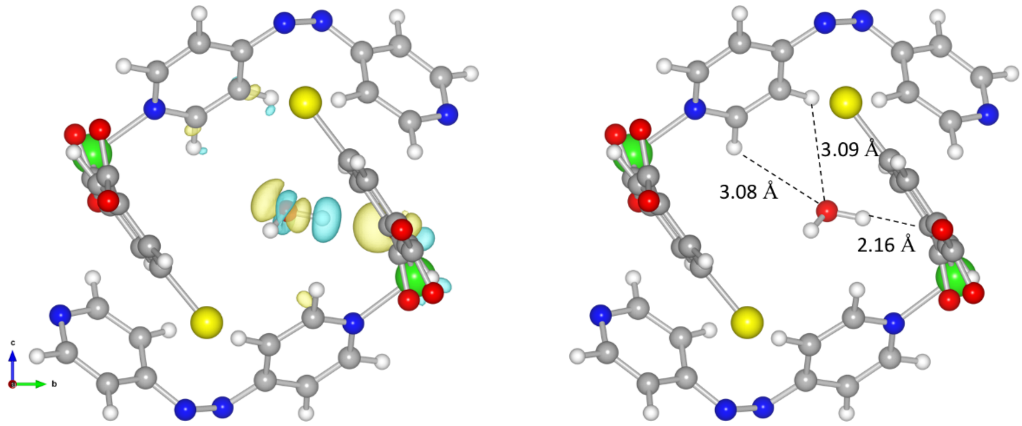

Figure 20. Optimal binding location of water in **1**. Distances drawn represent hydrogen bonds. The iso-surfaces represent charge redistribution upon binding, with blue being charge depletion and yellow being charge accumulation. The iso-level is set to 0.001 electrons per Å^3.

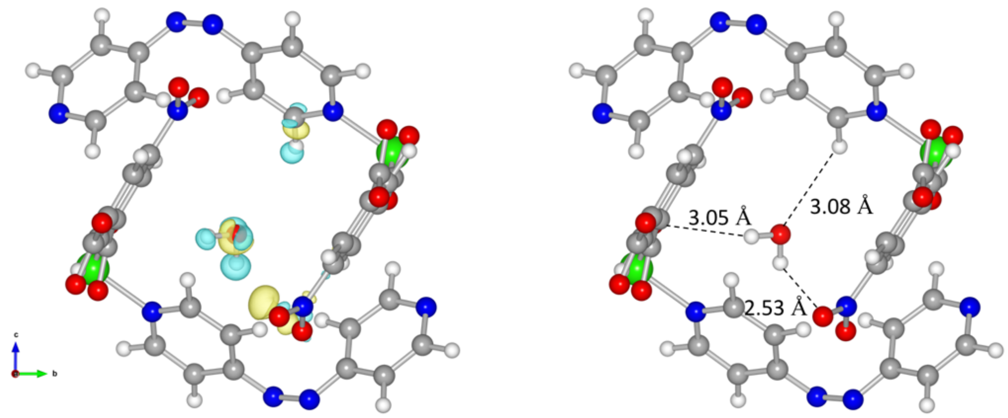

Figure 21. Optimal binding location of water in **2**. Distances drawn represent hydrogen bonds. The iso-surfaces represent charge redistribution upon binding with blue being charge depletion and yellow being charge accumulation. The iso-level is set to 0.001 electrons per Å^3.

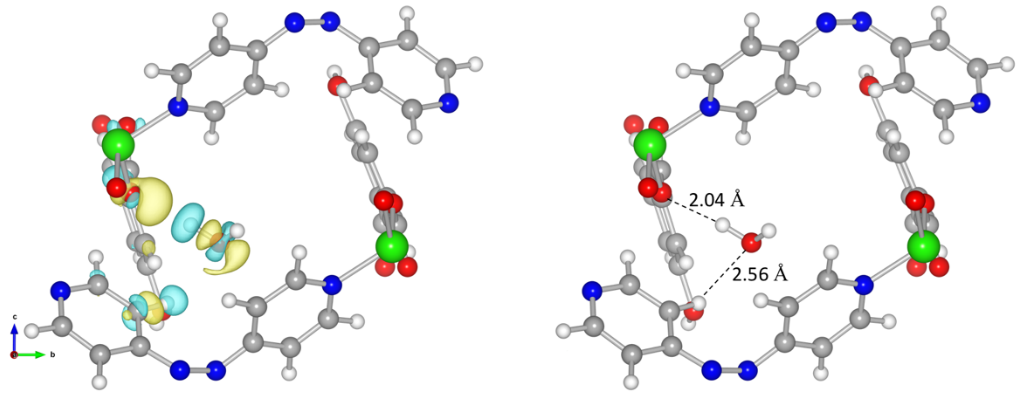

Figure 22. Optimal binding location of water in **3**. Distances drawn represent hydrogen bonds. The iso-surfaces represent charge redistribution upon binding with blue being charge depletion and yellow being charge accumulation. The iso-level is set to 0.001 electrons per Å^3.

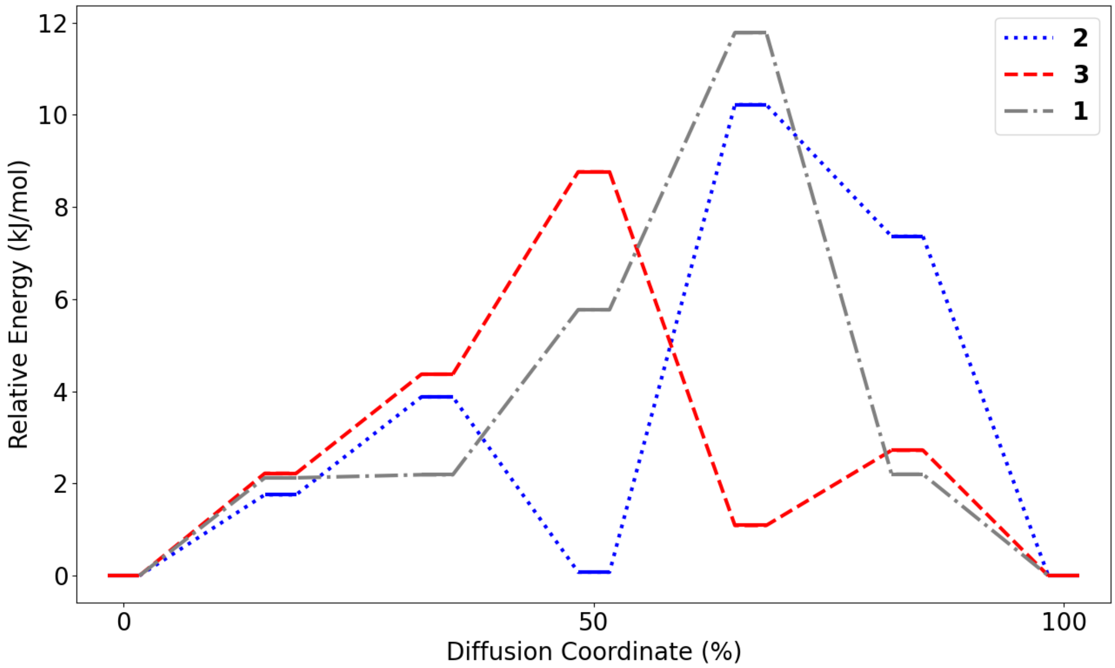

Figure S23. Diffusion barriers of a water molecule through the three structures. The diffusion pathways are from the optimal binding location to the identical location one cell over along the pore axis.

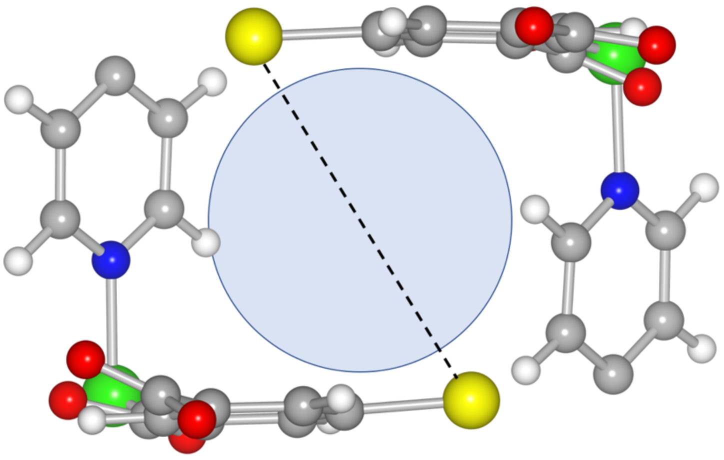

Figure S24. Depiction of the two planes used to sample the water potential energy landscape. The first plane (1) is circular and lies in the plane of the page. This plane constitutes the cross-section of the channel. The second plane (2) runs along the channel, between the substituent groups, and is perpendicular to the first plane, i.e. it sticks out of the page plane.

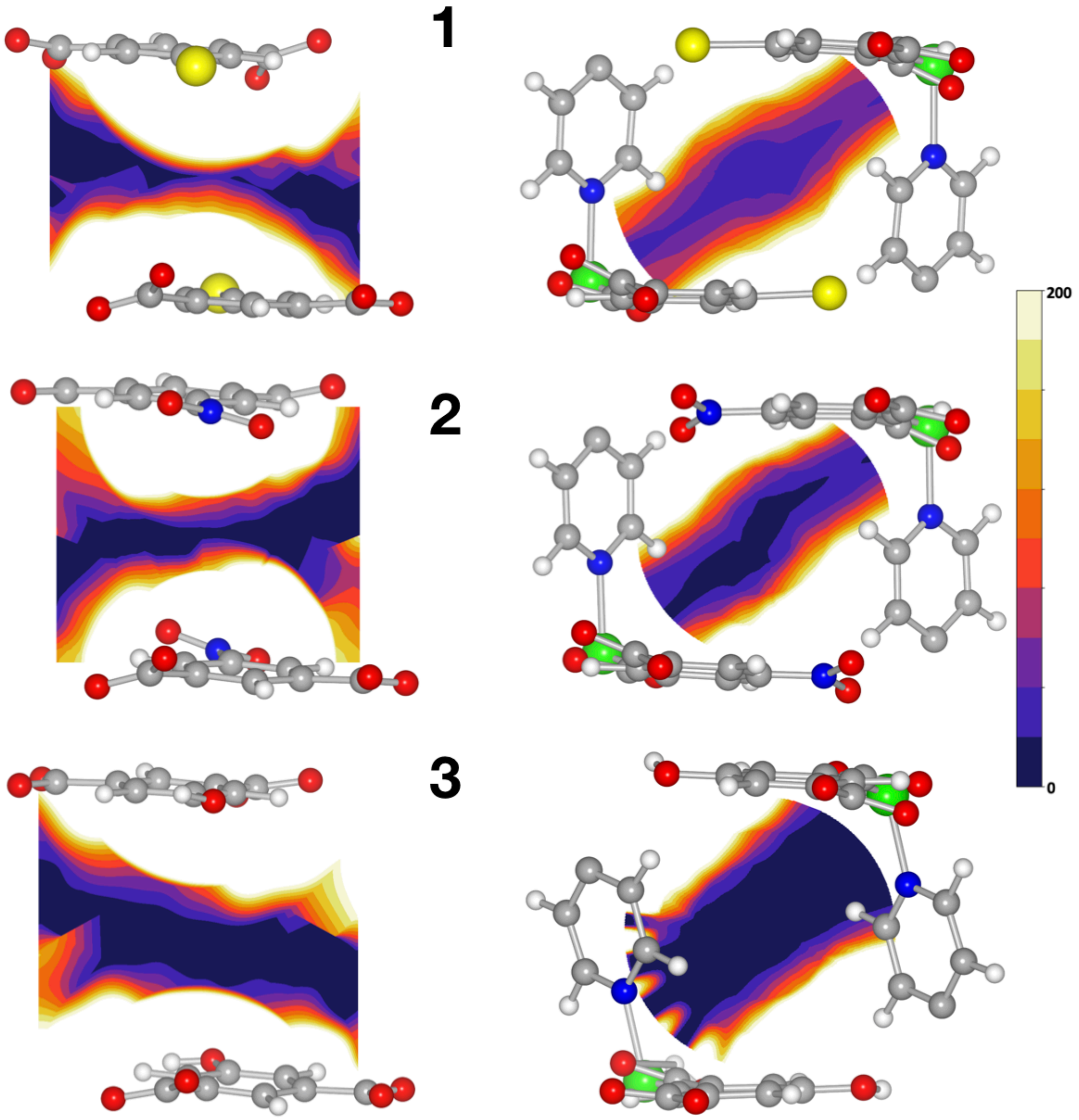

Figure S25. Potential energy landscape of water in structures **1, 2,** and **3**. The two planes are defined in Figure S19. The color bar is in kJ/mol. Also see Figure S21. The lowest potential energy value on those surfaces across all three systems was taken to be the zero-point for the color bar (the lowest point is located on the circular disk in **3**).

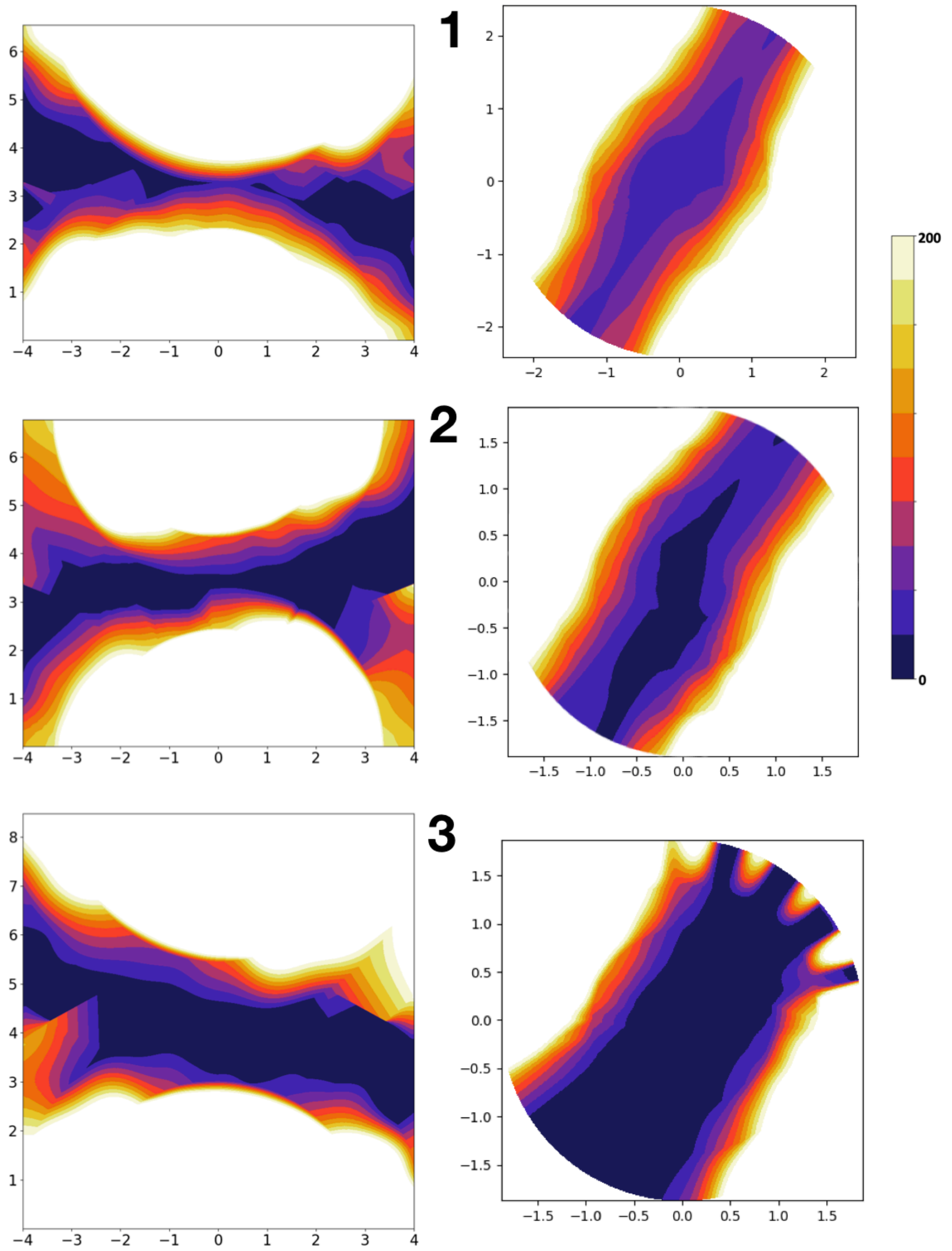

Figure S26. Potential energy landscapes in structures **1, 2,** and **3**. The two planes are defined in Figure S19. Both axes are in angstrom. The color bar is in kJ/mol. Also see Figure S20.

Table S1. Crystallographic data and structure refinement for **1**, [Zn(bia)(bphy)].

**Compounds**	**1**, [Zn(bia)(bphy)]
Identification code	2244649
Empirical formula	$C_{18}H_{13}BrN_4O_4Zn$
Formula weight	494.60
Temperature/K	100.00
Crystal system	monoclinic
Space group	$P2_1/n$
a/Å	10.2111(4)
b/Å	11.2281(4)
c/Å	18.9933(7)
α/°	90
β/°	95.9770(10)
γ/°	90
Volume/Å^3	2165.77(14)
Z	4
ρcalcg/cm^3	1.517
μ/mm^{-1}	3.007
F(000)	984.0
Radiation	MoKα (λ = 0.71073)
2Θ range for data collection/°	5.408 to 55.064
Index ranges	-13 ⩽ h ⩽ 13, -14 ⩽ k ⩽ 14, -24 ⩽ l ⩽ 24
Reflections collected	38635
Independent reflections	4979 [R_{int} = 0.0244, R_{sigma} = 0.0161]
Data/restraints/parameters	4979/0/253
Goodness-of-fit on F^2	1.076
Final R indexes [I>=2σ (I)]	R1[a] = 0.0393, wR2[b] = 0.0984
Final R indexes [all data]	R1[a] = 0.0439, wR2[b] = 0.1008
Largest diff. peak/hole / e Å^{-3}	1.88/-1.13

[a] $R_1=\Sigma||F_o|-|F_c||/\Sigma|F_o|$. [b] $wR_2= |\Sigma w(|F_o|^2-|F_c|^2)|/\Sigma|w(Fo^2)^2|^{1/2}$

Table S2. Unit cell parameters of **3** determined from *ab initio* calculations.

**Compounds**	**3**, [Zn(hia)(bphy)]
Empirical formula	$C_{18}H_{14}N_4O_5Zn$
Formula weight	431.69
Crystal system	monoclinic
Space group	$P2_1/n$
a/Å	10.3787
b/Å	11.4462
c/Å	18.2898
α/°	90
β/°	96.8656
γ/°	90
Volume/Å^3	2157.20

Table S3. Crystallographic atomic coordinates for the unit cell of [Zn(hia)(bphy)] (**3**).

Atom	x (Å)	y (Å)	z (Å)	Atom	x (Å)	y (Å)	z (Å)
Zn1	0.038033	0.365534	0.419293	C39	0.548647	0.202314	0.177619
Zn2	0.962926	0.637345	0.582883	C40	0.451832	0.795603	0.823168
Zn3	0.463021	0.862944	0.082871	C41	0.033298	0.71628	0.26688
Zn4	0.538138	0.1347	0.919296	C42	0.965038	0.284725	0.732752
C1	0.78063	0.37074	0.452373	C43	0.465011	0.215585	0.232765
C2	0.220798	0.62804	0.548632	C44	0.533255	0.783954	0.766878
C3	0.720847	0.872185	0.048623	C45	0.109299	0.618205	0.251634
C4	0.280753	0.129653	0.952404	C46	0.885001	0.380886	0.746123
C5	0.669194	0.34174	0.495524	C47	0.384967	0.119415	0.246104
C6	0.331595	0.655437	0.504639	C48	0.609281	0.882008	0.751623
C7	0.831646	0.844785	0.004633	C49	0.105911	0.518536	0.294475
C8	0.1693	0.158731	0.995534	C50	0.889022	0.480902	0.703986
C9	0.541973	0.361723	0.46283	C51	0.389025	0.019404	0.203955
C10	0.459111	0.639829	0.537708	C52	0.605937	0.981688	0.794468
C11	0.959158	0.860387	0.037704	C53	0.974644	0.005555	0.272793
C12	0.042091	0.13876	0.962813	C54	0.027582	0.997293	0.727367
C13	0.434896	0.327795	0.49772	C55	0.527478	0.503043	0.227404
C14	0.565561	0.673726	0.502086	C56	0.474647	0.494649	0.772796
C15	0.065603	0.826518	0.002067	C57	0.88684	0.100883	0.260863
C16	0.934988	0.172646	0.997695	C58	0.115175	0.902134	0.739004
C17	0.455152	0.277072	0.568277	C59	0.615085	0.598197	0.239057
C18	0.544548	0.719318	0.430289	C60	0.386874	0.399292	0.760864
C19	0.044578	0.780939	0.930262	C61	0.9074	0.200083	0.30395
C20	0.955201	0.223357	0.068264	C62	0.093746	0.802749	0.696301
C21	0.58277	0.258805	0.601614	C63	0.593706	0.697567	0.196347
C22	0.416517	0.732549	0.396236	C64	0.407474	0.300089	0.803939
C23	0.916542	0.767672	0.896219	C65	0.090518	0.119665	0.370802
C24	0.0828	0.241646	0.101622	C66	0.911114	0.884121	0.629356
C25	0.689881	0.289747	0.56552	C67	0.411111	0.616185	0.129361
C26	0.310128	0.702479	0.433194	C68	0.590564	0.380577	0.870794
C27	0.810162	0.797736	0.933183	C69	0.078244	0.016849	0.330341
C28	0.189941	0.210712	0.065543	C70	0.923216	0.986576	0.670179
C29	0.301544	0.341828	0.455695	C71	0.423151	0.513753	0.170197
C30	0.698887	0.663329	0.544814	C72	0.578262	0.48339	0.830336
C31	0.198928	0.836904	0.0448	H1	0.525622	0.405471	0.410205
C32	0.801635	0.158546	0.95567	H2	0.476285	0.599274	0.591282
C33	0.953616	0.601023	0.362658	H3	0.97634	0.900935	0.091285
C34	0.046298	0.401628	0.63814	H4	0.025765	0.095037	0.910175
C35	0.546354	0.0987	0.138159	H5	0.37233	0.250523	0.595752
C36	0.453641	0.899249	0.862672	H6	0.625503	0.745546	0.401375
C37	0.951863	0.704663	0.323163	H7	0.125529	0.754744	0.901333
C38	0.048624	0.298013	0.677585				

Table S4. Structural analysis of phases in [Zn(bia)(bphy)] (**1**), [Zn(nia)(bphy)] (**2**) and [Zn(hia)(bphy)] (**3**).

	d_a (Zn···Zn) Å	d_b (Zn···Zn) Å	Torsion angle (C-N-N-C) °	H bond lengths ($d_{N\cdots O}$, Å)		H bond angles ($\angle_{N-H\cdots O}$, °)	
	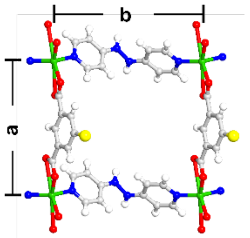		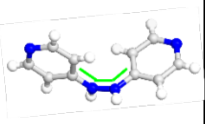	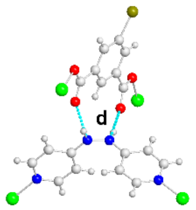		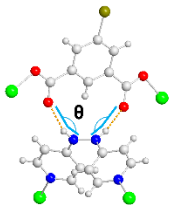	
**1**	10.211(1)	11.228(1)	98.5(4)	2.832(3)	2.861(4)	154.0(2)	167.9(2)
**2**	10.185(1)	11.104(1)	95.9(3)	2.864(3)	2.857(3)	152.2(2)	171.2(2)
**3**	10.379	11.446	103.6	2.830	2.894	171.6	175.9

12. References

(1) Launay, J. P.; Tourrel-Pagis, M.; Lipskier, J. F.; Marvaud, V.; Joachim, C. Control of Intramolecular Electron Transfer by a Chemical Reaction. The 4,4'-Azopyridine/1,2-Bis(4-Pyridyl)Hydrazine System. *Inorg. Chem.* **1991**, *30* (5), 1033–1038.

(2) Liu, X.; Lin, R.; Zhang, J.; Chen, X. Xiao-Min Liu, Rui-Biao Lin, Jie-Peng Zhang, and Xiao-Ming Chen. Low-dimensional porous coordination polymers based on 1, 2-bis (4-pyridyl) hydrazine: from structure diversity to ultrahigh CO2/CH4 selectivity. *Inorg. Chem.* **2012**, *51* (10), 5686-5692.

(3) Sheldrick, G. M.; Bruker, A. X. S. Inc., Madison, WI, 2000;(b) GM Sheldrick. *Acta Crystallogr., Sect. A Fundam. Crystallogr* **2015**, *71*, 3–8.

(4) Krause, L.; Herbst-Irmer, R.; Sheldrick, G. M.; Stalke, D. Comparison of Silver and Molybdenum Microfocus X-Ray Sources for Single-Crystal Structure Determination. *J. Appl. Crystallogr.* **2015**, *48* (1), 3–10.

(5) Dolomanov, O. V.; Bourhis, L. J.; Gildea, R. J.; Howard, J. A. K.; Puschmann, H. OLEX2: A Complete Structure Solution, Refinement and Analysis Program. *J. Appl. Crystallogr.* **2009**, *42* (2), 339–341.

(6) Sheldrick, G. M. Crystal Structure Refinement with SHELXL. *Acta Crystallogr. Sect. C Struct. Chem.* **2015**, *71* (Md), 3–8.

(7) Bezrukov, A. A.; O'Hearn, D. J.; Gascón-Pérez, V.; Darwish, S.; Kumar, A.; Sanda, S.; Kumar, N.; Francis, K.; Zaworotko, M. J. Metal-Organic Frameworks as Regeneration Optimized Sorbents for Atmospheric Water Harvesting. *Cell Reports Phys. Sci.* **2023**, *4* (2), 101252.

(8) Kresse, G.; Furthmüller, J. Efficient Iterative Schemes for Ab Initio Total-Energy Calculations Using a Plane-Wave Basis Set. *Phys. Rev. B - Condens. Matter Mater. Phys.* **1996**, *54* (16), 11169–11186.

(9) Kresse, G.; Joubert, D. From Ultrasoft Pseudopotentials to the Projector Augmented-Wave Method. *Phys. Rev. B* **1999**, *59* (3), 1758–1775.

(10) Berland, K.; Cooper, V. R.; Lee, K.; Schröder, E.; Thonhauser, T.; Hyldgaard, P.; Lundqvist, B. I. Van Der Waals Forces in Density Functional Theory: A Review of the VdW-DF Method. *Reports Prog. Phys.* **2015**, *78* (6), 66501.

(11) Langreth, D. C.; Lundqvist, B. I.; Chakarova-Käck, S. D.; Cooper, V. R.; Dion, M.; Hyldgaard, P.; Kelkkanen, A.; Kleis, J.; Kong, L.; Li, S. A Density Functional for Sparse Matter. *J. Phys. Condens. Matter* **2009**, *21* (8), 84203.

(12) Thonhauser, T.; Cooper, V. R.; Li, S.; Puzder, A.; Hyldgaard, P.; Langreth, D. C. Van Der Waals Density Functional: Self-Consistent Potential and the Nature of the van Der Waals Bond. *Phys. Rev. B* **2007**, *76* (12), 125112.

(13) Thonhauser, T.; Zuluaga, S.; Arter, C. A.; Berland, K.; Schröder, E.; Hyldgaard, P. Spin Signature of Nonlocal Correlation Binding in Metal-Organic Frameworks. *Phys. Rev. Lett.* **2015**, *115* (13), 136402.

(14) Henkelman, G.; Uberuaga, B. P.; Jónsson, H. A Climbing Image Nudged Elastic Band Method for Finding Saddle Points and Minimum Energy Paths. *J. Chem. Phys.* **2000**, *113* (22), 9901–9904.

(15) Dubbeldam, D.; Calero, S.; Ellis, D. E.; Snurr, R. Q. RASPA: Molecular Simulation Software for Adsorption and Diffusion in Flexible Nanoporous Materials. *Mol. Simul.* **2016**, *42* (2), 81–101.

(16) Horn, H. W.; Swope, W. C.; Pitera, J. W.; Madura, J. D.; Dick, T. J.; Hura, G. L.; Head-Gordon, T. Development of an Improved Four-Site Water Model for Biomolecular Simulations: TIP4P-Ew. *J. Chem. Phys.* **2004**, *120* (20), 9665–9678.

(17) Mayo, S. L.; Olafson, B. D.; Goddard, W. A. DREIDING: A Generic Force Field for Molecular Simulations. *J. Phys. Chem.* **1990**, *94* (26), 8897–8909.

(18) Rappe, A. K.; Casewit, C. J.; Colwell, K. S.; Goddard, W. A. I. I. I.; Skiff, W. M. UFF, a Full Periodic Table Force Field for Molecular Mechanics and Molecular Dynamics Simulations. *J. Am. Chem. Soc.* **1992**, *114* (25), 10024–10035.

(19) Wilmer, C. E.; Kim, K. C.; Snurr, R. Q. An Extended Charge Equilibration Method. *J. Phys. Chem. Lett.* **2012**, *3* (17), 2506–2511.
